# Poly(ionic liquid) Ionomers Help Prevent Active Site Aggregation, in Single-Site Oxygen Reduction Catalysts

**DOI:** 10.1021/acscatal.4c01418

**Published:** 2024-05-07

**Authors:** Silvia Favero, Alain Li, Mengnan Wang, Fayyad Uddin, Bora Kuzuoglu, Arthur Georgeson, Ifan E. L. Stephens, Maria Magdalena Titirici

**Affiliations:** † Department of Chemical Engineering, Imperial College London, SW7 2AZ London, U.K.; ‡ Department of Materials, Imperial College London, SW7 2AZ London, U.K.

**Keywords:** ionomer, poly(ionic liquid), anion exchange ionomer, oxygen reduction reaction, active site aggregation

## Abstract

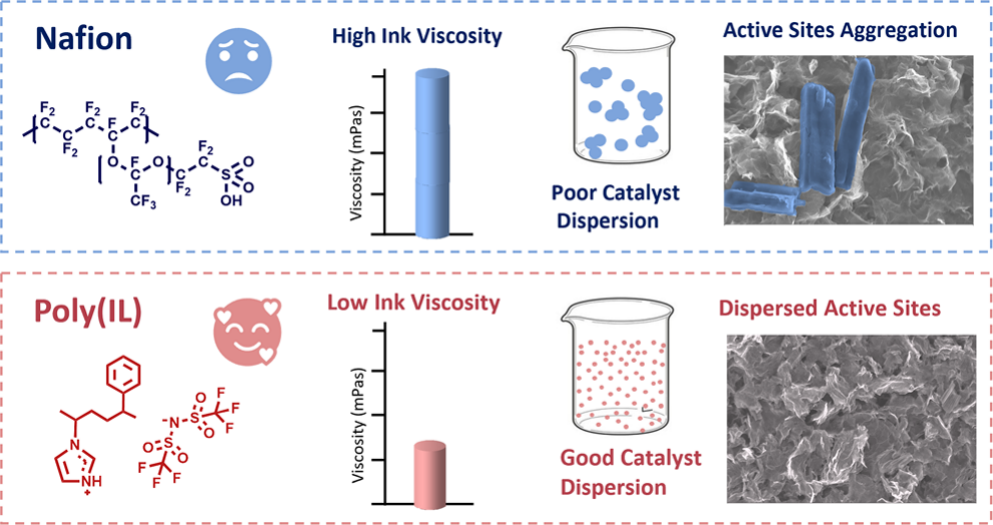

Anion exchange membrane fuel cells (AEMFCs) can produce clean electricity without the need for platinum-group metals at the cathode. To improve their durability and performance, most research investigations so far have focused on optimizing the catalyst and anion exchange membrane, while few studies have been dedicated to the effect of the ionomer. Herein, we address this gap by developing a poly(ionic liquid)-based ionomer and studying its effect on oxygen transport and oxygen reduction kinetics, in comparison to the commercial proton exchange and anion exchange ionomers Nafion and Fumion. Our study shows that the choice of ionomer has a dramatic effect on the morphology of the catalyst layer, in particular on iron aggregation. We also observed that the quality of the catalyst layer and the degree of iron aggregation can be correlated to the rheological properties of the catalyst ink. Moreover, this work highlights the impact of the ionomer on the resistance to oxygen transport and reports improved oxygen diffusion compared to Nafion, for poly(ionic liquid)s with fluorinated anions. Finally, the performance of the catalyst–ionomer layer for oxygen reduction was tested with a rotating disc electrode (RDE) and a gas diffusion electrode (GDE). We observed dramatic differences between the two configurations, which we attribute to the different morphologies of the catalyst layer. In summary, our study highlights the dramatic and overlooked effect of the ionomer and the limitations of the RDE in predicting fuel cell performance.

## Introduction

Anion exchange membrane fuel cells (AEMFCs) have received increasing attention as an alternative to their proton exchange membrane counterpart, due to the high oxygen reduction activity of transition metal catalysts in alkaline electrolytes. Recently, AEMFCs have obtained significant performance improvement, reaching power densities higher than 2 W cm^–2^.^1−6^ Such high performances have been achieved mainly via the optimization of the oxygen reduction catalyst and membrane structure. While both have been thoroughly investigated, less interest has been dedicated to the role of the ionomer.

The primary role of an ionomer is to bind the catalyst layer and provide ionic conductivity, but it can also have a significant impact on other fundamentals aspects of the catalyst layer, such as water management, gas transport, and electrode morphology. The vast majority of research studies in the literature, focusing on oxygen reduction catalysts in alkaline conditions, measure the activity using a rotating disc electrode (RDE) and employ the commercially available proton-conducting ionomer Nafion, sometimes with the protons exchanged with the cation prior to adding the catalyst.^7^ While the universal use of a standard ionomer allows easier comparison of catalytic activity, Nafion or similar acidic ionomers could potentially act as a barrier to the transport of hydroxide ions and can mask the true kinetic activity of the catalyst of interest.^8^ Only a very small number of RDE studies in alkaline conditions use alkaline ionomers,^9−12^ mostly employing the ionomer Tokuyama AS-4, the production of which is now discontinued. To the best of our knowledge, no study has been published comparing the performance of ionomers on oxygen reduction catalysts in the RDE. Only one similar report has been published looking at the effect of the ionomer on Pt/C for the hydrogen oxidation reaction.^8^ This work by Jervis et al. has shown that anion exchange ionomers (AEIs) consistently yield poorer electrode morphology, with large catalyst agglomerates compared to Nafion, yielding an artificially lower ECSA. Nevertheless, the catalyst layers obtained with the AEI yielded higher H_2_ oxidation kinetics than those containing Nafion, putatively due to improved OH^–^ transport.^8^ That study showed how H_2_ oxidation activity can be underestimated when using a proton-conducting ionomer (PEI) in alkaline conditions, and we conjecture that ionomers may also impose similar constraints on the activity of oxygen reduction catalysts.

Additionally, the geometry of the RDE cells is significantly different from that of real devices. In particular, the current density that can be achieved is much lower, as it is limited by oxygen solubility in the electrolyte. Therefore, although the study of ionomers with rotating disc electrodes can provide valuable insight on the ionomer–catalyst interaction on a kinetics level, it may lead to incorrect conclusions regarding transport effects and the optimum ionomer loading.

In an ideal scenario, the catalyst layer should be optimized comprehensively, taking into account the catalyst–ionomer interactions and measuring the performance in a membrane electrode assembly. A few ink formulation studies have shown that the nature of the ionomer, the choice of solvent, and the ionomer loading can have major effects on the morphology of the deposited electrode and ultimately on the performance of the fuel cell.^13−21^ These studies focus almost exclusively on proton exchange ionomers and platinum-based catalysts, illustrating the need and potential to optimize the ink composition for transition metal-based catalysts in alkaline conditions. However, membrane electrode assemblies are time-consuming and difficult to optimize, and the absence of established testing conditions and of a commercial standard anion exchange membrane makes comparison of the literature results extremely challenging. In this context, gas diffusion electrodes (GDEs) could offer an ideal intermediate to study the effect of ionomer nature and ink formulation on the catalyst layer in more realistic conditions, while minimizing interlaboratory variations in operating conditions. Several gas diffusion electrode geometries have been proposed, including a vertically sandwiched cell, developed by Arenz and co-workers,^22^ a commercially available cell by Gaskatel, a modified floating electrode apparatus,^23^ and a vertically sandwiched geometry by Cherevko and co-workers.^24^ In all cases, it was demonstrated that the gas diffusion electrode can offer high oxygen mass transport, allowing to access the intrinsic activity of the catalyst over a wide potential range (0.6–0.9 V vs RHE), in agreement with results obtained with the RDE at low overpotentials and MEA at high overpotentials.^25^ An interlab comparison has additionally shown that reliable and comparable measurements are possible with all the mentioned setups.^26^

In this work, we compare the activity of a commercially available iron single-site catalyst (iron phthalocyanine) when using the state-of-the-art proton exchange ionomer (Nafion), a commercially available anion exchange ionomer (Fumion), and in-house developed poly(ionic liquid)-based ionomers. We first synthesized and characterized the poly(ionic liquid)s, based on a copolymer of styrene and vinyl imidazole, with an NTF2 fluorinated anion (poly(St-*co*-VImNTF2)). We then used rheology studies and dynamic light scattering (DLS) to study the catalyst–ionomer interactions for the selected ionomers, measuring the ink viscosity and aggregate size. Scanning electron microscopy (SEM) was used to compare the morphology of the catalyst layers obtained with the various ionomers, via spray coating on a carbon paper or drop casting on a glassy substrate. The catalyst layers were finally tested for oxygen reduction in both a half-cell rotating disc electrode and a gas diffusion electrode (GDE). The results show significant differences between the two measurements, highlighting the different roles of mass transport and the importance of testing novel catalysts and ionomers in GDE setups.

## Results and Discussion

### Polymer Synthesis and Characterization

**Figure 1:**
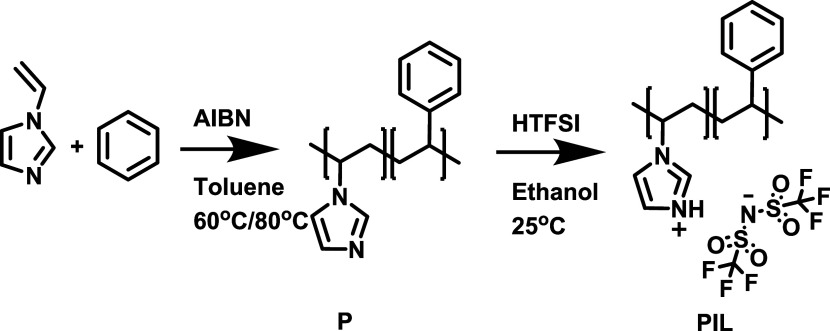
Schematic of the polymerization and ion exchange reactions, to produce the copolymer poly*-co*-styrene-vinylimidazole (P) and the poly(ionic liquid) polymer poly*-co*-styrene-vinylimidazolium-trifluorosulfonilimide (PIL).

Copolymers of styrene and vinyl imidazole were synthesized and purified using different conditions summarized in Table 1 and are referred to as P1, P2, and P3. The polymers were then subjected to an ion exchange with HTFSI, to obtain the corresponding poly(ionic liquid)s PIL1, PIL2, and PIL3 (Figure 1). The structure of the poly(ionic liquid)s was chosen to provide hydrophobicity (by the styrene monomers) and oxygenophilicity (provided by the fluorinated anion). To study the relative importance of these parameters and the effect of the polymer composition, the initial monomer ratio was varied in the different synthesis. NMR spectra (Supporting Information, Figure S1) confirm the expected structure of the polymers and poly(ionic liquid)s and the successful ion exchange. Elemental analysis was employed to study the styrene-to-vinyl imidazole ratio after polymerization, and the results are shown in Table 1. Polymers 1 and 2 present a similar St:VIm ratio of 1:2.2 and 1:2.3, respectively, while polymer 3 shows a much higher imidazolium content. Since polymers 2 and 3 were obtained from the same synthesis, varying uniquely the isolation and purification steps, these results show the preference of the monomers to form a homopolymer. Due to the limited solubility of the obtained polymers in suitable solvents, it was not possible to determine the molecular weight with gel permeation chromatography. Nevertheless, qualitative information can be drawn for the solubility of the polymers and the viscosity of polymeric solutions. From these observations, it can be inferred that polymer 1 has a much smaller molecular weight compared to the other two, followed by polymer 2 and polymer 3. NMR and elemental analysis show the successful incorporation of TFSI anions after ion exchange. This yields a library of poly(ionic liquid)s with the same monomers and varying morphology, allowing to isolate the effect of molecular weight and monomer composition on the performance of the ionomer.

**Table 1:** Synthesis Conditions for the Polymers Poly-*co*-St-VIm

	synthesis parameters							resultant polymer	
polymer	synthesis	molar ratio of Vim/St	VIm (mL)	toluene (mL)	AIBN (mg)	temperature (°C)	precipitation	St/VIm ratio	molecular weight
P1/PIL1	a	7.5:1	5	100	9	60	dropped in cold diethyl ether	2.2:1	low
P2/PIL2	b	4:1	4.4	20	16	80	toluene-soluble fraction dropped in cold diethyl ether	2.3:1	medium
P3/PIL3							precipitate from the synthesis in toluene isolated and washed in diethyl ether	1:4.8	high

Due to the low molecular weight, the inks obtained with the polymer and poly(ionic liquid) 1 displayed low viscosity and weak ability to bind the catalyst particle, making the deposition of the catalyst layer challenging, inhomogeneous, and sometimes irreproducible. Where meaningful electrochemical results could be obtained, they are shown in the Supporting Information, but further characterization and discussion herein will focus on polymers 2 and 3.

**Figure 2:**
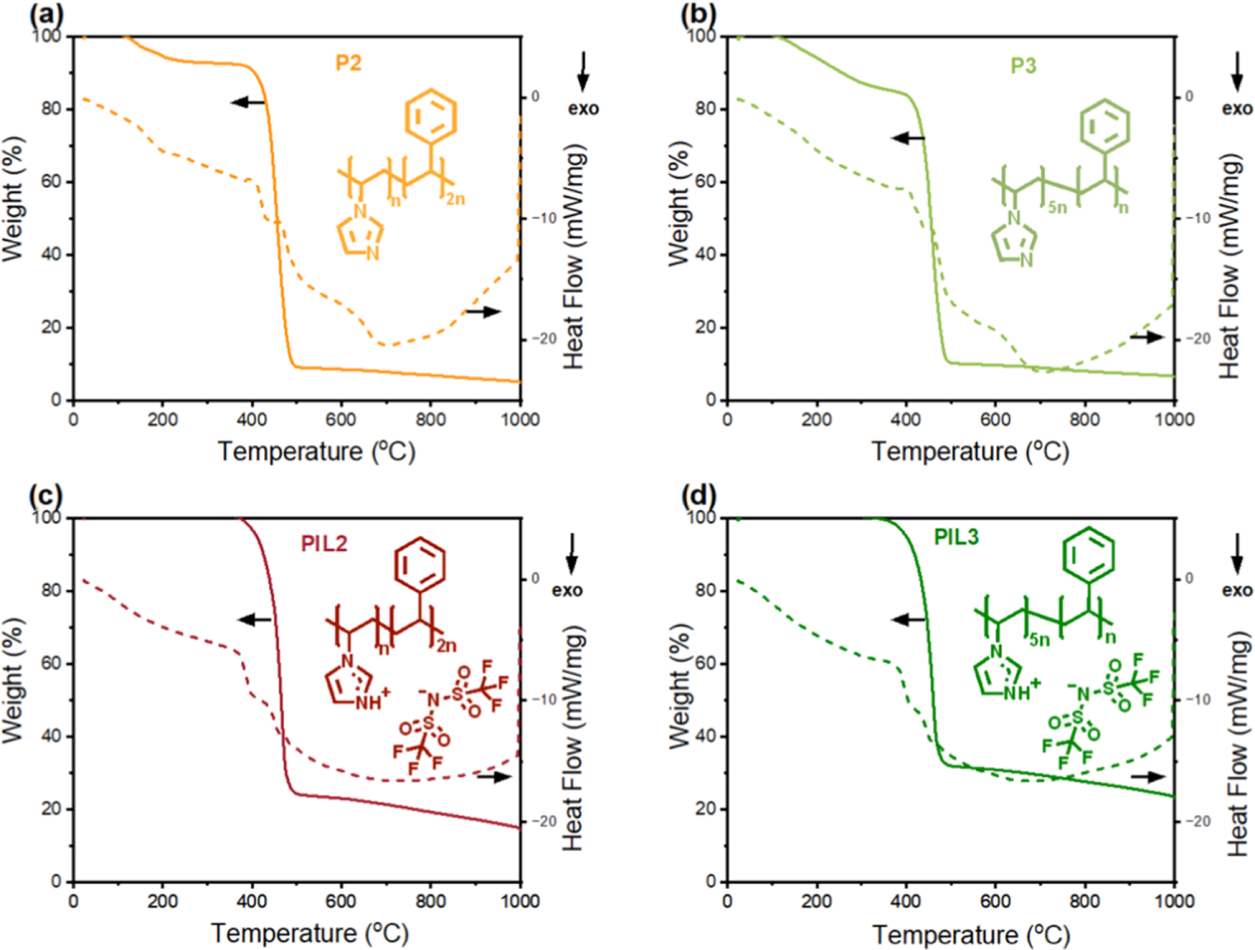
Simultaneous thermogravimetric analysis (solid line) and differential scanning calorimetry (dotted line) of the polymers P2 and P3 and on the poly(ionic liquid)s PIL2 and PIL3.

Simultaneous thermogravimetric analysis and differential scanning calorimetry were used to characterize the thermostability of the obtained polymers P2, P3, PIL2, and PIL3 (Figure 2). Both polymers P2 and P3 are stable up to 120 °C, while the degradation onset increases to over 350 °C after the ion exchange. As such, all polymers are stable in the typical operating temperature range of AEM fuel cells. All the samples showed a big exothermic degradation peak at high temperature, while they do not reveal endothermic melting peaks.

### Characterization of the Ink and Catalyst Layer

Scanning electron microscopy was used to observe the effect of the ionomer and solvent on the structure of the catalyst layer, either drop-cast on a glass substrate (to recreate the film present in RDE measurements) or spray-coated on carbon paper (following the same procedure as for GDE measurements). Iron phthalocyanine (Fe(II)PC) was chosen as a model FeN_4_ catalyst for this study. For the case of the commercial ionomers Nafion and Fumion, 50% IPA/water and ethanol were used as ink solvents to observe the effect of the solvent on the catalyst morphology. The poly(ionic liquid)s PIL2 and PIL3 were found to be insoluble in both water and isopropanol; therefore, results are shown only for ethanol.

**Figure 3:**
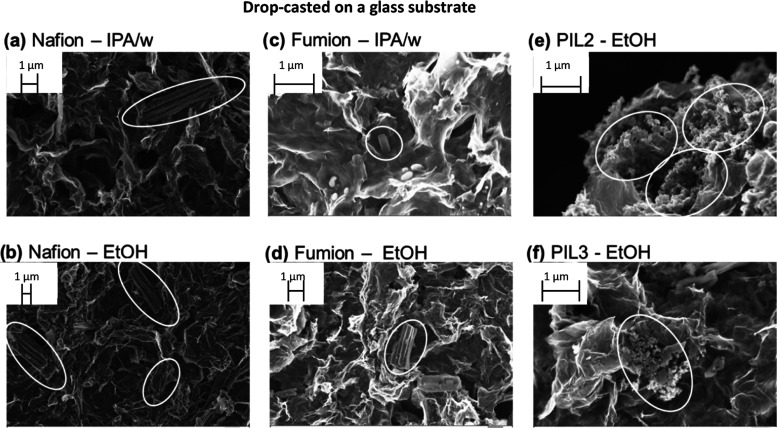
SEM images of the catalyst ink drop-cast on a glass substrate; all the inks contain 4 mg/mL FePC/G catalyst and ionomer with I/C ratio = 1. (a) Ink containing Nafion as an ionomer, solvent is 50%IPA in water, (b) ink containing Nafion as an ionomer, solvent is ethanol, (c) ink containing Fumion as an ionomer, solvent is 50%IPA in water, (d) ink containing Fumion as an ionomer, solvent is ethanol, (e) ink containing the in-house synthesized PIL2 as an ionomer, solvent is ethanol, and (f) ink containing the in-house synthesized PIL3 as an ionomer, solvent is ethanol.

**Figure 4:**
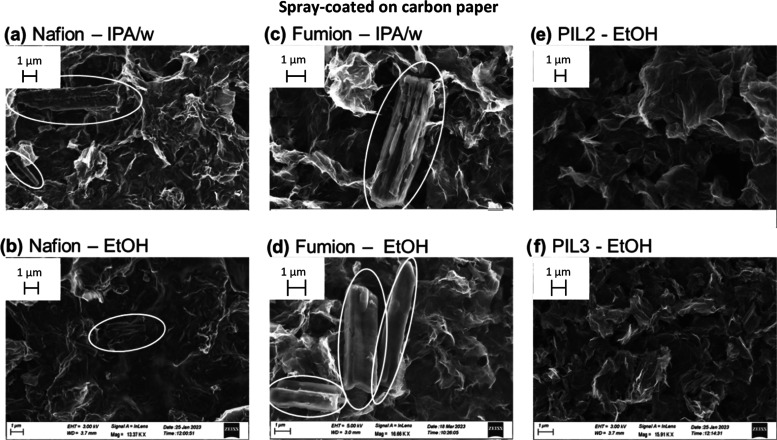
SEM images of the catalyst ink spray-coated on carbon paper; all the inks contain 4 mg/mL FePC/G catalyst and ionomer with I/C ratio = 1. (a) Ink containing Nafion as an ionomer, solvent is 50%IPA in water, (b) ink containing Nafion as an ionomer, solvent is ethanol, (c) ink containing Fumion as an ionomer, solvent is 50%IPA in water, (d) ink containing Fumion as an ionomer, solvent is ethanol, (e) ink containing the in-house synthesized PIL2 as an ionomer, solvent is ethanol, and (f) ink containing the in-house synthesized PIL3 as an ionomer, solvent is ethanol.

On a macroscopic scale, the choice of ionomer and solvent was found to have a small effect on the morphology of the catalyst layer spray-coated on carbon paper (Supporting Information, Figure S3). On the contrary, the ionomer had a more significant effect on the drop-cast samples, with the poly(ionic liquid) inks, particularly PIL3, generating the most uniform layers. The same can also be observed on a macroscopic level with an optical microscope (Figure S15). More interestingly, Figures 3 and 4 show that the catalyst layers obtained with Nafion and Fumion ionomers present cylindrical aggregates (highlighted with white circles) with an average diameter of around 1 μm and several micrometers in length, independently of the deposition substrate or technique. EDX elemental mapping (Supporting Information, Figures S4–S6) shows that these structures are rich in carbon, nitrogen, and iron, suggesting that they originate from aggregation of iron phthalocyanine, likely by π–π stacking. The formation of such aggregates is undesired, as it reduces accessibility to the active sites and catalyst utilization. FePC aggregation has already been observed and it was reported to depend on the surface area of the carbon support, the FePC/support mass ratio, and the molecule–carbon interaction.^27^ Here, for the first time, we report that the maximum loading before aggregation can also be increased by the choice of ionomer. In fact, the hereby-synthesized poly(ionic liquid)s were found to be superior in dispersing the active sites, as the iron-rich aggregates were not observed when using PIL2 or PIL3 as ionomers, neither on carbon paper nor glass.

However, the poly(ionic liquid) inks drop-cast on glass showed spherical aggregates, 50–100 nm in diameter, which were found to be rich in sulfur and oxygen and are therefore assigned to the ionomer. These spherical ionomer aggregates were instead not observed when the ink was spray-coated on carbon paper.

The reason for the ionomer aggregation being observed only on the glass sample was not further investigated, but we propose that it could originate from the difference in drying time between the deposition techniques. For the case of drop-casting on glass, typically, a few microliters of droplet is deposited and left to dry at ambient temperature, allowing the ionomer more time and space to rearrange. On the other side, spray coating on carbon paper is performed on a heated plate (maintained at 50 °C), and a much thinner layer is deposited and left to dry before spraying more catalyst, limiting the ionomer ability to rearrange and potentially leading to a more uniform distribution when spray-coating on carbon paper, rather than drop-casting on glass. Another possibility is that the different morphologies could be due to the intrinsic hydrophobicity and geometry of the substrate, leading to significant differences in the water contact angle on carbon paper (130°)^28^ and glassy carbon (60°).^29^

**Figure 5:**
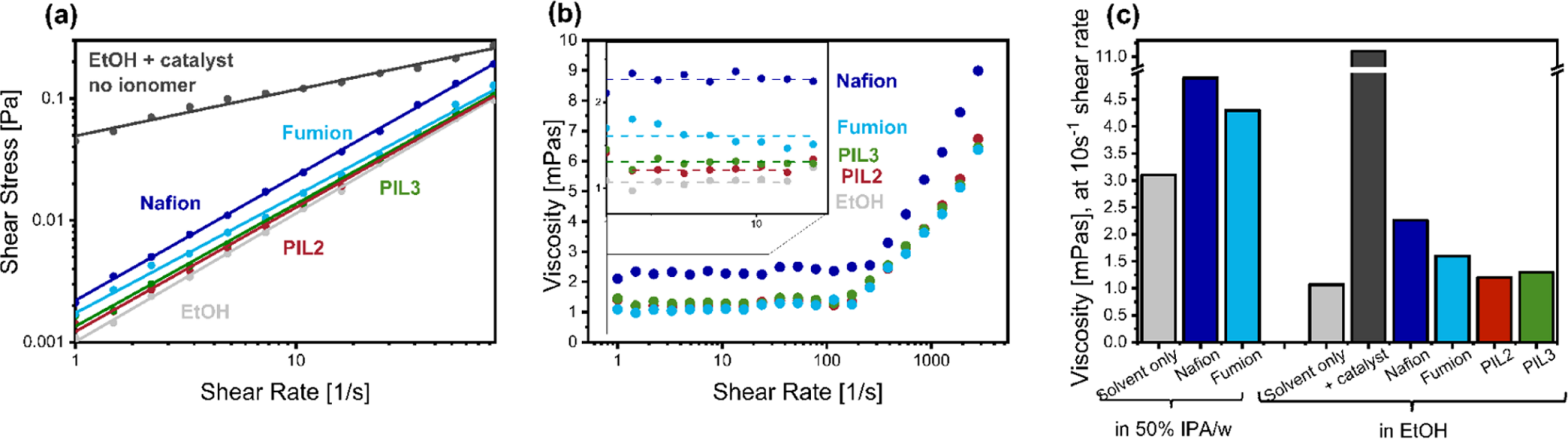
Rheology Investigation of the catalyst inks. All the inks contain 4 mg/mL FePC/G catalysts, with various ionomers in an I/C ratio of 1. The solvent is either ethanol of 50% wt isopropanol in water. (a) Shear stress as a function of shear rate at low shear rates, for the inks containing various ionomers and ethanol as a solvent, the lines represent a linear fitting. (b) Viscosity as a function of shear rate (with a zoom-in at low shear rate and linear fitting), for the inks containing various ionomers and ethanol as a solvent. (c) Viscosity at 10 s^–1^ shear rate for various catalyst inks.

The formation of the cylindrical FePC aggregates was further investigated by studying the catalyst inks in the presence of different ionomers, with rheology and dynamic light scattering. Rheology studies were performed in rotational cylinder geometry. To directly compare the effect of different ionomers, inks were prepared in ethanol with the same catalyst and ionomer concentration, and the results are shown in Figure 5. In the absence of any ionomer, the catalyst–solvent ink was found to have a strong shear-thinning behavior up to 100 s^–1^ (Supporting Information, Figure S7), which has been previously attributed to the breakdown and rearrangements of agglomerates induced by the liquid flow.^30^ On the contrary, all of the ionomer-containing samples exhibited lower viscosity and Newtonian behavior, for shear rates up to 100 s^–1^, indicating electrosteric stabilization of the catalyst agglomerates. All the samples showed a weak shear-thickening behavior at higher shear rates, which can be attributed to the breakup of agglomerates and consequent increase in the effective volume fraction of the particles.^31^ In all the shear rate ranges investigated, Nafion exhibited the highest viscosity, while the poly(ionic liquid)s showed the lowest. These observations indicate that the poly(ionic liquid)s are superior in dispersing the catalyst particle and could explain the lack of FePC aggregates observed with SEM.

**Figure 6:**
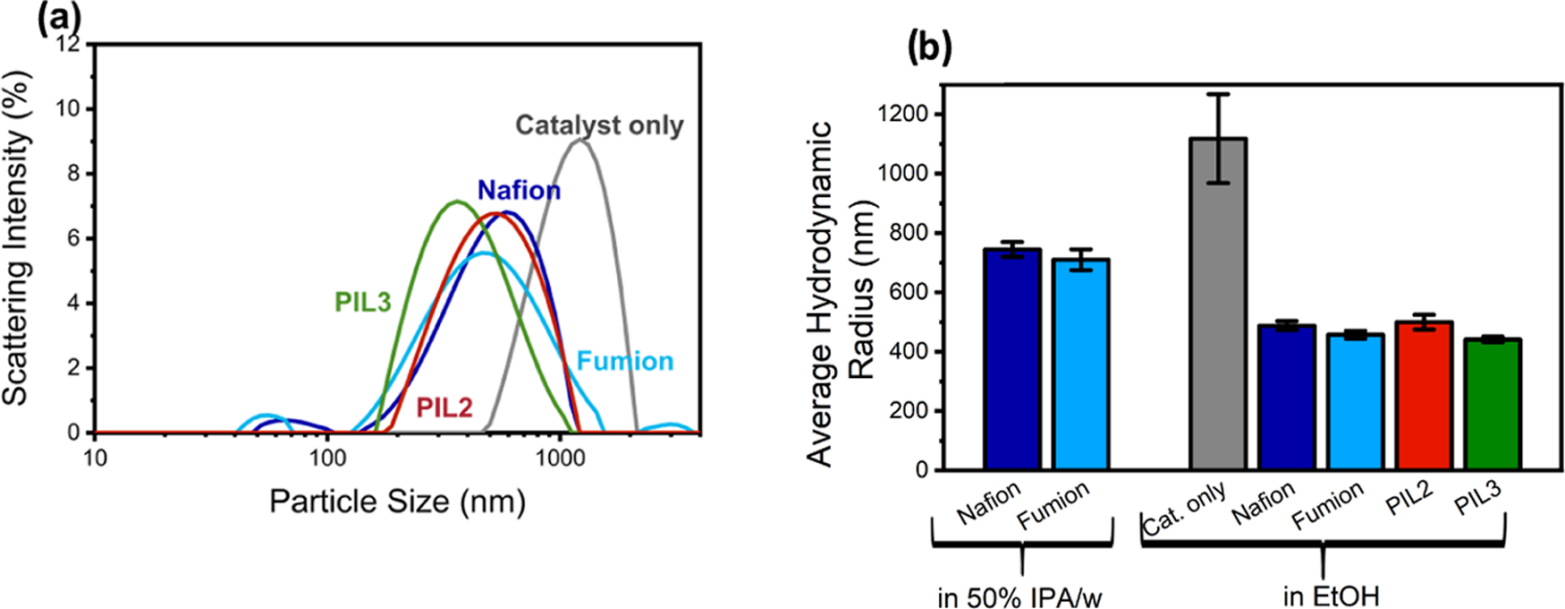
Dynamic light scattering of the catalyst inks, diluted 100 times. After dilution, the inks contained 0.04 mg of catalyst and 0.04 mg of ionomer per mL of ink. (a) Scattering intensity as a function of particle size for the inks in ethanol and (b) average hydrodynamics radius, averaged over a minimum of three runs of 10 measurements each.

Dynamic light scattering was also used to estimate the size of the catalyst agglomerates. All the inks were freshly prepared for the measurement, left stirring when not in use, and ultrasonicated for 10 min before the measurement. Most often, DLS measurements are performed on the sole solvent and ionomer, in the absence of the catalyst, because the high catalyst concentrations used in the inks limit the application of this technique. This approach can assist in the choice of solvent for polymer dispersion but fails to provide information regarding ionomer–catalyst interactions. Secanell and co-workers have reported that dilution of the catalyst ink does not significantly affect DLS measurements and that 1:10 and 1:100 dilution leads to an underestimation of the particle size of only 16 and 19%, respectively.^32^ Therefore, DLS measurements were performed on the catalyst-containing inks that were diluted 100 times. The results are shown in Figure 6, and more details are given in the Supporting Information, Figure S8 and Table S1. As expected, the catalyst ink in the absence of an ionomer is very unstable and the particle size varies between repeats, with the average hydrodynamics radius ranging between 930 and 1230 nm. After the addition of any ionomer, the dispersion becomes more stable, the measurements are more reproducible, and the average particle size is lower. Noticeably, the average particle size is lower in ethanol (400–500 nm) than in IPA/water (700–750 nm), for both the commercial ionomers tested. The ionomer of choice was found to have less impact compared to the solvent, with all the ionomers yielding aggregates with size ranging from 400 to 500 nm. In particular, PIL3 provided the lowest particle size, in accordance with the superior catalyst layer morphology revealed by SEM.

### Electrochemical Results

The performance of the poly(ionic liquid)s as alkaline ionomers was initially tested using a rotating disc electrode, with FePC/G as a model FeN_4_ catalyst. As already mentioned, the commercially available Nafion and Fumion were selected as proton-conducting and anion-conductive ionomers, respectively; to provide a comparison against the performance of the hereby-synthesized poly(ionic liquid) ionomers. Fumion KOH activation was investigated and was found to have negligible effects on the performance of the catalyst (Supporting Information, Figure S12b). Fumion and Nafion were tested in both ethanol and 50% IPA/water, and only small differences were observed between the two, so results in the main paper are shown only for IPA/water inks, which were found to be easier to drop-cast (Supporting Information, Figure S12a). The ionomer-to-catalyst ratio (I/C) was initially optimized using Nafion (Supporting Information, Figure S11). An I/C ratio of 0.25 was found to be insufficient, while small differences were observed above 0.5; an I/C ratio of 1 was finally selected and used for all the measurements, unless otherwise stated.

Inks containing P1 or PIL1 as ionomers were challenging to deposit and yielded inhomogeneous catalyst films, probably due to the short chain length of the polymer and its consequently poor binding ability. The ORR performance of the P1 and PIL1-containing catalyst layer was similarly poor (Supporting Information, Figure S9) and will therefore not be discussed further.

**Figure 7:**
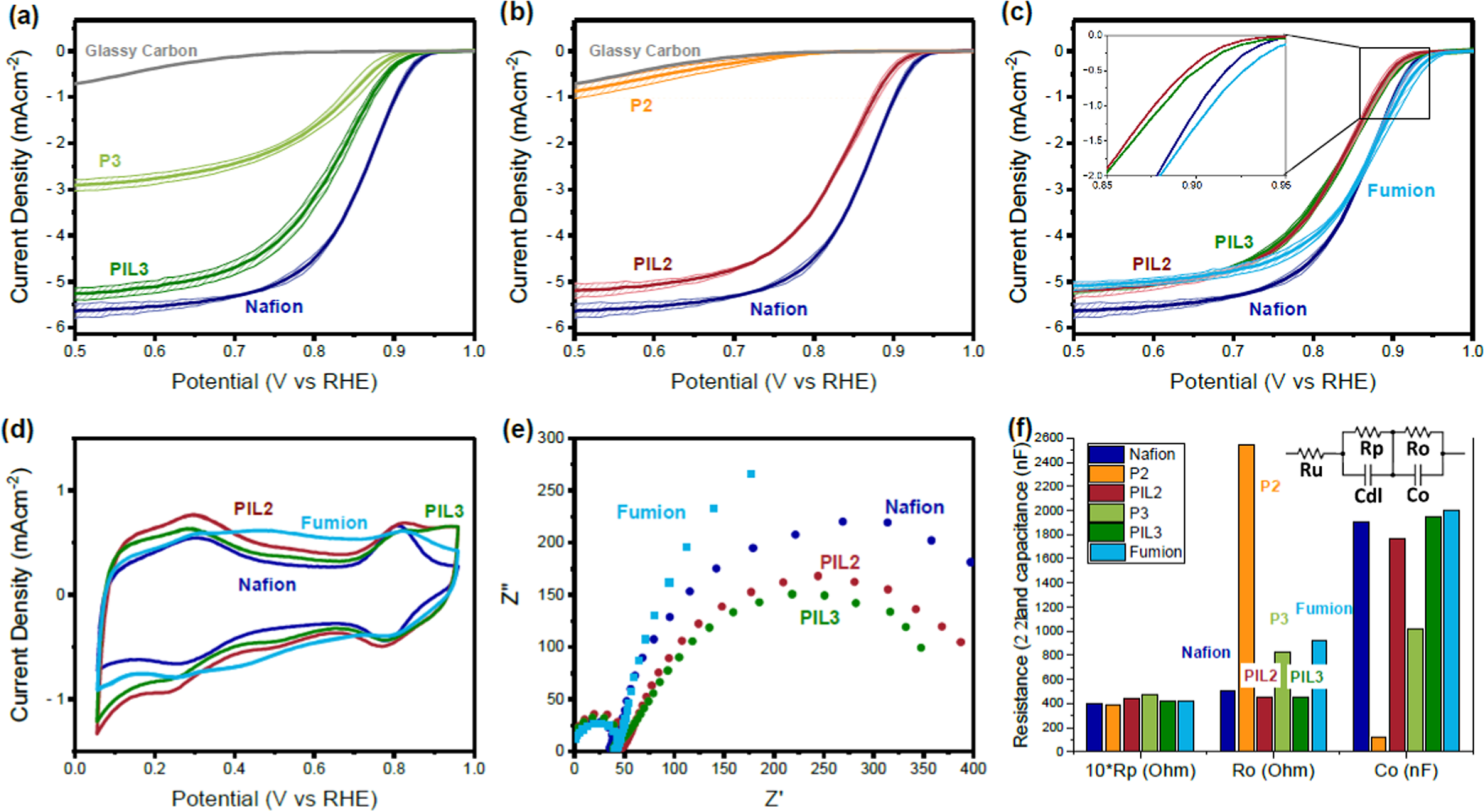
Rotating disc electrode results. All data were collected using FePC/G as a catalyst, with the addition of the ionomer shown in the figure and an I/C ratio of 1. All data were obtained at 25 °C using a Hg/HgO reference electrode and graphite rod counter electrode. (a–c) Cathodic linear sweep voltammogram (from 1 to 0.5 V vs RHE) in oxygen-saturated 0.1 M KOH, at a rotational speed of 1600 rpm, 10 mV/s scan rate, with various ionomers as indicated in the graphs: Nafion in blue (solvent 50%IPA in water), Fumion in light blue (solvent 50%IPA in water), PIL2 in red (solvent ethanol), and PIL3 in green (solvent ethanol). All samples were preconditioned with 50 cycling voltammograms in nitrogen (scanning between 0.1 and 0.9 V vs RHE), before saturating the electrolyte with oxygen, removing bubbles on the surface, and collecting LSV data. (d) Cyclic voltammogram of FePC/G with the addition of Nafion (blue), Fumion (light blue), PIL2 (red), and PIL3 (green) ionomers. Data were collected in nitrogen-saturated 0.1 M KOH, in static conditions with a scan rate of 50 mV/s, in a potential range of 0.1 to 0.9 V vs RHE; the scan shown here is the 50th collected. (e) Electrochemical impedance spectroscopy of FePC/G with the addition of Nafion (blue), Fumion (light blue), PIL2 (red), and PIL3 (green) ionomers. Data were collected at 0.8 V vs RHE in a frequency range of 0.1 Hz to 1 M Hz, in oxygen-saturated 0.1 M KOH at a rotational rate of 1600 rpm. (f) Results of impedance fitting with the equivalent circuit detailed in the Supporting Information. From left to right are shown the polarization resistance, the resistance to oxygen transport, and the capacitance term of oxygen storage.

As can be observed in Figure 7a,b, the polymers before ion exchange P2 and P3 display remarkably poor oxygen reduction performance. This is likely attributed to the low ionic conductivity or oxygen transport properties. To test the second hypothesis, we performed electrochemical impedance spectroscopy (Figure 7e) at the mixed kinetic-diffusion region (0.8 V vs RHE) and fitted the results to an equivalent circuit to obtain an estimate of the oxygen transport resistance.

There is still debate regarding the appropriate interpretation of impedance spectroscopy results in the rotating disc electrode, but there is a widespread consensus that qualitative results can be obtained from the low-frequency semicircle regarding oxygen transport.^33−36^ More details regarding the choice of equivalent circuit are provided in the Supporting Information.

Figure 7f shows the results of the impedance fitting. As can be observed, the polarization resistance is similar for all of the ionomers tested. On the other side, significant differences are observed in the resistance to oxygen transport. In particular, P2 has the highest resistance, explaining the low ORR performance. P3, which is richer in vinylimidazolium monomers, displays improved oxygen transport and ORR performance. After the ion exchange and the introduction of a fluorinated, oxygenophilic anion, both PIL2 and PIL3 display drastically reduced oxygen transport resistance, lower than that offered by either Nafion or Fumion. This improved oxygen transport and ionic conduction translate in a drastic improvement in the ORR performance between the polymers and the poly(ionic liquid)s (Figure 7a,b). The difference between P2 and P3 is no longer observed between PIL2 and PIL3, likely indicating that oxygen transport is not the limiting factor for the poly(ionic liquid)s.

Despite the improvement, PIL2 and PIL3 still display poorer ORR performance compared to Nafion or Fumion. Since oxygen reduction is a complex process, there could be several factors explaining this behavior. In this work, we considered oxygen transport, ionomer loading, kinetic effects, and electrode architecture, as described below.

Starting with oxygen transport, low oxygen permeability in the poly(ionic liquid) ionomer could explain the lower performance. However, impedance spectroscopy data showed that the poly(ionic liquid)s exhibit the highest oxygen transport among the ionomers selected. Therefore, we discarded this possibility.

Second, we considered the possibility that the selected ionomer loading for the polyIL could be insufficient to fully cover the catalyst and yield a uniform layer. However, no improvement was observed when doubling the I/C ratio to 2 (Supporting Information, Figure S10), so this hypothesis was also rejected.

Another hypothesis is that the ionomers could interact with the catalysts by blocking the active sites by competitive adsorption or interacting with reaction intermediates, thus modifying the reaction kinetics. To monitor changes in the number of electrochemically accessible active sites and in the binding energy of the ORR intermediates, we observed the size and position of the high-potential cyclic voltammetry peak. This peak is generally agreed to originate from desorption of the *OH intermediate from the iron center,^37^ and as a result, the size of this peak is a measure of the amount of active sites and its position is a measure of the *OH binding energy. Since the position of this peak does not change between PIL3 and Nafion (Figure 7d), it can be concluded that the ionomers tested do not strongly interact with the adsorbed intermediates. On the contrary, PIL2 seems to shift the peak to slightly higher potentials, which indicates a weaker *OH bond and should lead to higher ORR kinetics. For the case of Fumion, the high-potential peak also appears shifted to slightly higher potentials, likely due to the improved *OH transport offered by the anion-conductive ionomer. This also explains the higher activity of the Fumion-containing catalyst in the kinetic region (Figure 7c). For the Fumion sample, the low-potential peak is composed of two convoluted peaks, one of which we attribute to the ionomer. Further discussion on this topic can be found in Figure S12c.

Finally, we attribute the poor performance of the poly(ionic liquids) to the morphology of the catalyst layer and in particular to the poor ionomer distribution (as evidenced by Figure 3e,f) and the consequent lack of a reliable 3D network for the transport of water and hydroxyl ions. These conclusions are further supported by a loading study shown in Figure S15 and performed in the presence of Nafion and PIL2 ionomers. This study shows stronger loading sensitivity in the case of PIL2, which suggests poor accessibility to the active sites.

To summarize the results thus far, the addition of a fluorinated anion to the initial polymer drastically improves oxygen transport and overall ORR performance of the polymers. The so-obtained PIL2 and PIL3 showed lower oxygen transport resistance compared to those of both Nafion and Fumion. However, the performance of the catalyst with the poly(ionic liquid) ionomers still does not reach that of the commercial ionomers; we attribute this poorer performance to the fact that PIL2 and PIL3 aggregate in the catalyst layer, rather than dispersing homogeneously. As such, the performance of PIL2 and PIL3 should be improved when the catalyst ink is spray-coated rather than being drop-cast, as for the first case, no polymeric aggregates were observed under SEM. To test this hypothesis, the same ionomers were tested under the more realistic conditions of a gas diffusion electrode.

**Figure 8:**
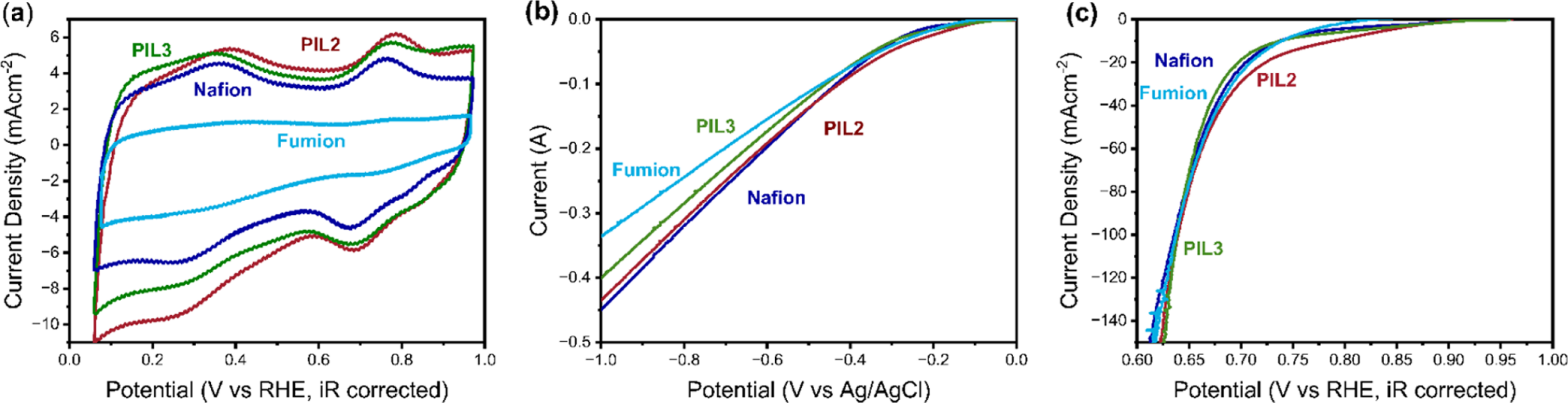
Gas diffusion electrode results of the FePC/G catalyst with the ionomer shown in the graphs in an I/C ratio of 1. Cyclic voltammogram of FePC/G with Nafion (blue), Fumion (light blue), PIL2 (red), and PIL3 (green). Data were collected in a gas diffusion electrode with a nitrogen-saturated 1 M KOH electrolyte, at a scan rate of 100 mV/s, between 0.05 and 0.95 V vs RHE. Displayed here is the 10th cycle. Linear sweep voltammogram of FePC/G with Nafion (blue), Fumion (light blue), PIL2 (red), and PIL3 (green). Data collected in a gas diffusion electrode with an oxygen-saturated 1 M KOH electrolyte, at a scan rate of 5 mV/s. Shown here are the data obtained after conditioning the electrode with 10 CV scans between 0 and −1 V vs Ag/AgCl in an O_2_-saturated electrolyte, at a scan rate of 5 mV/s. Linear sweep voltammogram of FePC/G with Nafion (blue), Fumion (light blue), PIL2 (red), and PIL3 (green) after iR correction. The ohmic resistance was calculated using impedance spectroscopy; it was found to be constant in the potential range considered and did not change before or after the measurement. The ohmic resistance was in the range of 1.5–2 Ω for all measurements. Data collected in a gas diffusion electrode with an oxygen-saturated 1 M KOH electrolyte, at a scan rate of 5 mV/s. Shown here are the data obtained after conditioning the electrode with 10 CV scans between 0 and −1 V vs Ag/AgCl in an O_2_-saturated electrolyte, at a scan rate of 5 mV/s.

Figure 8b,c shows the oxygen reduction performance before and after iR correction, respectively. These results are remarkably different from those observed with a rotating disc electrode, particularly for the case of the poly(ionic liquid)s. At high current density, PIL2 and PIL3 offer ORR performance within the experimental error of that of the commercial ionomer, while in the RDE, the poly(ionic liquid)s showed a lower performance compared to the commercial ionomer. Once again, since the catalyst, ionomers, and I/C ratios are the same, we hypothesize that the difference arises from the morphology of the catalyst layer. In particular, the SEM images showed that the poly(ionic liquid)s aggregate in spherical particles when drop-cast on a glass support (as is the case for the RDE), while they distribute homogeneously when spray-coated on a carbon support (as is the case for GDE testing). The aggregation of the ionomer could explain the low performance of PIL2 and PIL3 in the RDE and the better performance obtained in GDE tests.

At low current density, the poly(ionic liquid) PIL2 displays higher activity compared to the other ionomers. Since this difference is only observed in the kinetic region, we expect it to originate from a positive influence of PIL2 on the activity of the catalyst rather than from transport effects or from the morphology of the catalyst layer. To confirm the effect of the ionomer on the activity of the iron site, we can once again observe the position of the high-potential CV peak, which depends on the strength of the *OH bond. As it can be observed in Figure 8a, the high-potential CV peak for PIL2 appears at slightly more positive potential, than for Nafion or PIL3, indicating a weaker *OH bond and supporting the effect of PIL2 on the activity of the active site. Since PIL2 and PIL3 differ only in the relative monomer ratio, this activity enhancement can be attributed to the higher amount of the styrene monomer. Many effects could contribute to the positive influence of styrene, and further investigation would be necessary to confirm the influence of this monomer. Among these possibilities, we suggest that the hydrophobic nature of the styrene-rich PIL2 might be responsible for *OH destabilization and consequent activity enhancement. Weakening of the *OH bond in the absence of the hydrogen bonds with solvated water has already been reported on platinum.^38^

For the case of Fumion, the improved activity observed in the RDE does not translate to GDE measurements. The cyclic voltammogram of the Fumion-containing catalyst layer in the GDE reveals much lower double layer capacitance and peak size compared to the other ionomers. This indicates poor accessibility to the active sites, likely originating from FePC aggregation, which was much more severe in the spray-coated samples (Figure 4c,d) than for the drop-cast ones (Figure 3c,d)

**Figure 9:**
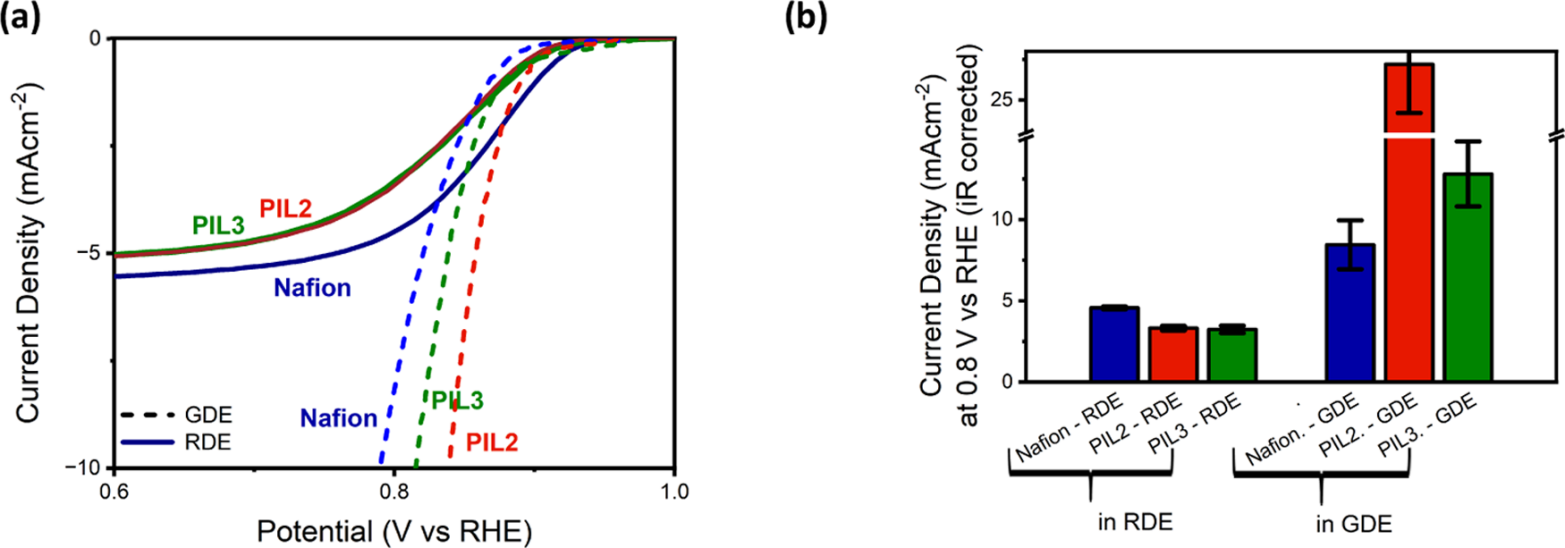
Comparison of oxygen reduction activity tested in a rotating disc electrode and gas diffusion electrode setup. All the measurements were taken with the FePC/G catalyst, with a catalyst-to-ionomer ratio of 1. (A) Linear sweep voltammograms. The full lines represent data collected with a rotating disc electrode, in oxygen-saturated 0.1M KOH, at a rotational speed of 1600 rpm, with a Hg/HgO reference electrode and graphite rod counter electrode. The dashed line represents data collected with a gas diffusion electrode setup, in oxygen-saturated 1M KOH, with a platinum counter electrode. (B) Current density at 0.8 V vs RHE, extracted from (a).

Finally, Figure 9 offers a visual comparison of the activity in the presence of the PIL2, PIL3, and Nafion ionomer, in the RDE and GDE. As previously reported, the performance of Nafion in the kinetic region is much higher in the RDE than in the GDE,^25^ with an apparent shift in the onset potential of more than 100 mV. On the contrary, results obtained with the poly(ionic liquid) ionomers in the RDE and GDE seem to coincide at low current densities. Although the trend would have to be confirmed with different catalysts, this observation suggests that poly(ionic liquid)s could be used in RDE measurements to obtain a more reliable estimation of the performance of catalysts in real devices.

## Conclusions

This study offers for the first time a direct comparison of the effect of the choice of ionomer on the oxygen reduction performance of an FeN_4_-based catalyst (FePC) in alkaline conditions. To this end, we selected commercially available proton-conductive (Nafion) and anion-conductive ionomers (Fumion), with a selection of hydrophobic and oxygenophilic poly(ionic liquid)s (PIL 1,2,3) hereby synthesized.

First, we successfully synthesized the poly(ionic liquid)s poly*-co*-(styrene)-(vinylimidazolium-trifluorosulfonilimide), with different chain lengths and monomer ratios (referred to as PIL1, PIL2, and PIL3), and we confirmed their chemical structure by NMR and elemental analysis.

The catalyst inks and catalyst layers obtained with the various ionomers were investigated using rheology measurements and dynamic light scattering. It was found that, independently of the deposition method used, the commercial ionomers caused FePC aggregation in cylindrical clusters of around 1 μm diameter. FePC aggregation was not observed when using the hereby-synthesized poly(ionic liquid) ionomers, in line with the fact that PIL-based catalyst inks displayed lower viscosity and aggregate size compared to their commercial ionomer counterparts.

The PIL ionomers were found to aggregate in spherical clusters when drop-cast on a glass substrate, while the same was not observed when the catalyst ink was spray-coated on a carbon paper. We presume that the difference originates from the deposition method and, in particular, from the volume of ink and time to dry but could also originate from specific interactions with the support. Independently of the origin, the ionomer aggregation led to poor performance of the PILs when tested in the RDE, while the performance in the GDE was comparable to that of the commercial ionomers. These observations highlight the importance of the catalyst layer morphology and how the performance of an ionomer can be controlled by its effect on the catalyst morphology rather than by its intrinsic properties.

Finally, when measured in a gas diffusion electrode, the oxygen reduction performance of FePC with PIL2 was superior to that of all other ionomers at low overpotentials. PIL2 was also found to shift the high-potential CV peak to more positive potentials, indicating weakening of the *OH bond and potentially explaining the improved kinetics. This effect is attributed to the hydrophobic nature of PIL2, which compared to PIL3 has a higher proportion of styrene monomer units.

## Materials and Methods

### Synthesis

Toluene, diethyl ether, azobis(isobutyronitrile) (AIBN), 1-vinylimidazole, and styrene were purchased from Sigma-Aldrich, of which the 1-vinylimidazole and styrene were purified with an alumina filter column and the rest were used as received.

Styrene and vinylimidazole copolymers were synthesized following a procedure reported by Zhang et al.^39^ In brief, desired amounts of 1-vinylimidazole (VIm), styrene (St), and AIBN (see Table 1) were dissolved in anhydrous toluene under an argon atmosphere in a Schlenk flask. Three freeze–pump–thaw cycles were performed to remove oxygen present in the reaction vessel. The mixture was then placed in an oil bath heated to 60 °C (synthesis a) or 80 °C (synthesis b) and left for approximately 12 h. Synthesis yielded a very low-molecular weight polymer, referred to as P1, that was recovered by dropping the solution in cold diethyl ether. The synthesis was then optimized by varying the monomer ratio and the relative amount of the solvent and initiator and by further purifying the reactants. Monomer purification and AIBN recrystallization were not found to have a significant effect on the polymerization, while temperature had the biggest effect. The optimized synthesis, referred to as synthesis b, was performed at 80 °C. After the polymerization in toluene, part of the resultant polymer had dropped at the bottom of the flask. This was recovered and termed P3, while the polymer dissolved in toluene is referred to as P2. All the so-obtained polymers (P1, P2, and P3) were dropped into cold diethyl ether solvent, resulting in a “sticky” orange precipitate. All the precipitates were washed with hexane and methanol, using a Soxhlet setup and dried under a vacuum at 80 °C for 24 h. The resultant polymers, poly(styrene-*co*-vinylimidazole), are indicated as P1, P2, and P3.

The poly(ionic liquid)s PIL1, PIL2, and PIL3 were obtained from the respective polymers (P1, P2, and P3), by ion exchange. The copolymers and excess TFSI were dissolved in ethanol, and the mixture was stirred at room temperature for 12 h using a magnetic stirrer. The ion-exchanged polymers were dropped into deionized water and washed five times, which produced a pale-white precipitate, poly(St-*co*-VImH+). The resultant poly(ionic liquid)s were left to dry under a vacuum at 40 °C for 24 h.

### Characterization

Simultaneous thermogravimetric analysis and differential scanning calorimetry were performed on the polymers P2 and P3 and on the poly(ionic liquid)s PIL2 and PIL3 using a Netzsch STA 449 F5 Jupiter. Experiments were performed in a temperature range of 30 to 1000 °C, using a heating rate of 20 °C/min, under a nitrogen flow rate of 40 mL/min.

Rheological measurements were performed by using an Anton Paar MCR 302 rheometer. All the measurements were conducted with a rotational cylinder geometry at 25 °C. Steady-state rheological data were collected at a steady state in increasing logarithmic shear rate steps from 1 to 2800 s^–1^. The samples tested included pure solvents (ethanol and 50% wt isopropanol in water), solvents with the catalyst (4 mg/mL), and solvents with the catalyst and ionomer (4 mg/mL catalyst +4 mg/mL ionomer).

Dynamic light scattering measurements were performed using a Malvern Zetasizer MicroV, to determine the aggregate size in the catalyst ink. Since the catalyst inks were too concentrated for the instrument specifications, they were diluted 100 times, yielding catalyst inks containing 0.04 mg of catalyst and 0.04 mg of ionomer per mL on solution. The inks were prepared fresh on the day of the measurements and stirred when not in use. Before each measurement, the ink was ultrasonicated for 10 min to ensure complete dispersion. Each measurement consisted of 10 runs of 10 s each. The measurements were repeated at least three times. In general, all of the inks were found to be stable for the duration of the experiment if tested right after the ultrasonication, with the exception of the ionomer-free sample.

1H nuclear magnetic resonance (H1 NMR) spectra were collected by using a Bruker Avance III HD 800 MHz NMR spectrometer. *d*_6_-DMSO was used as a deuterated solvent.

### Scanning Electron Microscopy (SEM)

Scanning electron microscopy (LEO Gemini 1252 FEG-SEM) was used to study the morphologies of samples operating at an accelerating voltage of 3 keV and working distance of around 3 mm. Before the measurement, the samples were secured onto the alumina sample holders by using conductive double-sided carbon tapes. For the sample drop-cast on the glass substrate, conductive liquid silver paint was used to create an effective conductivity path of the sample surface to prevent charge buildup.

### Optical Microscopy

The optical microscope VHX-7000 was used to collect images and profilometry of the RDE electrode.

### Elemental Analysis

Elemental analysis was carried out using energy-dispersive X-ray spectroscopy (EDX) integrated with the SEM machine. Accelerating voltage was increased to 10 keV and the working distance was increased to 10 mm before capturing the spectra.

### Electrochemical Measurements: RDE

For all the electrochemical tests, iron(II) phthalocyanine (FePC) was used as a model catalyst with graphene as a carbon support. FePC and graphene were ground in a mortar in 1:1 mass ratio. The resulting graphene-supported macrocycles were then used to prepare the inks. All the inks were prepared with a catalyst concentration of 4 mg/mL, and the synthesized polymers and Nafion were used as ionomers, with a catalyst/ionomer mass ratio (I/C) varying between 0.25 and 2. For all the tests involving Nafion, 50% wt IPA in water was used as the solvent, while the novel polymers were tested using ethanol as the solvent. All the inks were dispersed by 20 min of bath sonication and 10 min of probe ultrasonication.

The catalyst ink was spin-coated on a rotating disc electrode (5 mm-radius glassy carbon RDE, Metrohm, previously polished with a micropolish cloth and 0.05 μm alumina suspension), to obtain a loading of 0.14 mg/cm^2^. The electrodes were left to dry for 2 h before testing.

Electrochemical measurements were performed in a 3-electrode electrochemical cell, featuring a rotating disk working electrode, a graphite rod counter electrode, and a Hg/HgO reference electrode. The reference electrode was calibrated regularly, and all the potentials in this paper are reported vs RHE, for RDE testing. The calibration was performed in H_2_-saturated 0.1 M KOH, using a platinum RDE working electrode (from Metrohm), at 1600 rpm.

In a typical RDE experiment, the electrolyte (0.1 M KOH, Suprapur, Merch) was saturated with oxygen (99.9998% Ultrapure Plus, Air Products) and linear sweep voltammograms (LSV) were collected at a scan rate of 10 mV/s and rotational speed ranging from 400 to 2400 rpm. Cyclic voltammograms (CV) were collected in static, N2-saturated (99.99998% BIP Plus, Air Products) 0.1 M KOH at a scan rate of 50 mV/s. Finally, impedance spectroscopy data were collected in oxygen-saturated 0.1 M KOH, at a rotational speed of 1600 rpm, at 0.8 V vs RHE, using an amplitude of 1% of the applied voltage and measuring 10 data points per decade, in a frequency range of 0.1 Hz to 100 kHz. Details of fitting are provided in the Supporting Information.

All tests were repeated three times; reported in the paper are the average and the standard deviation (for LSV measurements).

### Electrochemical Measurements: GDE

PIL2 and PIL3 were further tested as ionomers in the gas diffusion electrode cell Flexcell PTFE, from Gaskatel. More details on the setup and testing protocol can be found in the preprint recently published by our group.^40^ Catalyst inks were prepared with the same composition as that used for RDE testing and an I/C ratio of 1. Inks containing Nafion were made of 4 mg/mL catalyst and 4 mg/mL Nafion in 50% IPA/water solvent. Inks containing PIL2 or PIL3 were made of 4 mg/mL catalyst and 4 mg/mL ionomer in ethanol. Similar to RDE testing, all the inks were dispersed by 20 min of bath sonication and 10 min of probe ultrasonication.

The catalyst inks were consequently sprayed on a hydrophobic gas diffusion layer (Sigracet 39BB, by Dioxide Materials), using a spray gun. In all cases, a circular area of 2 cm^2^ was sprayed to obtain a catalyst loading of 1 mg_catalyst_/cm^2^. Ag/AgCl was used as a reference electrode, and a platinum rod served as a counter electrode. All measurements were obtained in 1 M KOH (Suprapur, Merch). Oxygen (99.9998% Ultrapure Plus, Air Products) or nitrogen (99.99998% BIP Plus, Air Products) was flowed on the gas side and bubbled in the electrolyte, at a flow rate of 200 mL/min. All measurements were conducted at room temperature. Cyclic voltammograms were obtained in nitrogen, at a scan rate of 50 mV/s; linear sweep voltammograms were obtained in oxygen at a scan rate of 5 mV/s. Impedance data were collected in oxygen at a range of 0 to −0.4 V vs Ag/AgCl, the range over which the ohmic drop showed negligible change.

## Supporting Information Available

NMR spectra of the poly(ionic liquid)s; optical microscopy and profilometry images of the catalyst layer; SEM and EDX images of the catalyst layer; rheology analysis of the catalyst inks; dynamic light scattering of the catalyst aggregated in the catalyst ink; LSV data for the optimization of the I/C ratio; LSV data for the optimization of Fumion activation; LSV data of the effect of the catalyst loading; and details on the equivalent circuits for the fitting of impedance spectroscopy data (PDF)
