# Supplementary material for: Poly(ionic liquid) Ionomers Help Prevent Active Site Aggregation, in Single-Site Oxygen Reduction Catalysts
Source: ACS Catal. 2024 May 7;14(10):7937–48. doi: 10.1021/acscatal.4c01418 (PMC11106738; doi:10.1021/acscatal.4c01418)
Supplement: Supplementary file 1 — cs4c01418_si_001.pdf [file cs4c01418_si_001.pdf]

Supporting Information

Poly(ionic liquid) ionomers help prevent active site aggregation, in single site oxygen reduction catalysts

Silvia Favero*[a], Alain Li [a], Mengnan Wang[a], Fayyad Uddin[a], Bora Kuzuoglu [a], Arthur Georgeson [a], Ifan E. L. Stephens [b], Maria Magdalena Titirici* [a]

* silvia.favero15@imperial.ac.uk

* m.titirici@imperial.ac.uk

[a] Department of Chemical Engineering, Imperial College London, SW7 2AZ, UK

[b] Department of Materials, Imperial College London, SW7 2AZ, UK

Figure S 1: Typical NMR spectra of the poly(ionic liquid)s poly-co-styrene-vynilimidazoleNTF2 (PIL1, PIL2, PIL3) 2
Figure S 2: Typical NMR spectra of the polymers poly-co-styrene-vynilimidazole (P1, P2, P3) 2
Figure S 3: SEM images of catalyst ink drop casted on a glass substrate (first two columns) or spray coated on carbon paper (third and fourth column). 3
Figure S 4: SEM image and EDX elemental mapping of an electrode area showing cylindrical aggregate typical of the Nafion and Fumion samples. 4
Figure S 5: SEM image and EDX elemental mapping of an electrode area showing round aggregate typical of the PIL2 and PIL3 samples drop casted on glass. 4
Figure S 6: SEM image and EDX elemental mapping 5
Figure S 7: Rheology of the catalyst inks. 6
Figure S 8: Dynamic light scattering of the catalyst inks, diluted 100 times. 7
Figure S 9: Comparison of the ORR performance of Nafion and the poly(ionic liquid)s ionomers. 8
Figure S 10: Optimization of Ionomer to Catalyst ratio for Nafion. 8
Figure S 11: Optimization of the ionomer to catalyst ratio for the poly(ionic liquid)s ionomers 8
Figure S 12: Further electrochemical studies of Fumion ionomer. 9
Figure S 13: Equivalent circuit for fitting of Impedance spectroscopy measurements 10
Figure S 14: Comparison of Impedance fitting parameters obtained with the 11
Figure S 15: Effect of catalyst loading on the oxygen reduction performance. a) shows the oxygen reduction performance of FePC/G with Nafion ionomer, at the loadings indicated in the legend. b) shows the same results, when using PIL2 as an ionomer. For all the measurements, an I:C ration of 1 was used 11
Figure S 16: Optical microscopy images of the RDE electrode with a drop-casted layer of 0.14 mg cm^{-2} FePC/G with the ionomers Nafion, PIL2 and PIL3, with an I:C ratio of 1. All the inks contained the following: 4mg/ml FePC/G, ionomer with I/C ratio=1. The solvent used was ethanol for PIL2, PIL3 and 50% isopropanol in water for Nafion. 14

1. NMR spectra

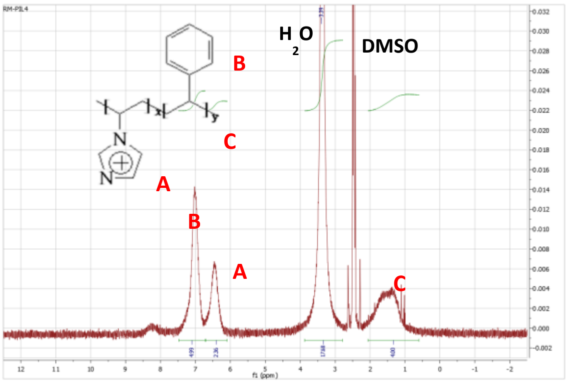

Figure S 1: Typical NMR spectra of the poly(ionic liquid)s poly-co-styrene-vynilimidazoleNTF2 (PIL1, PIL2, PIL3)

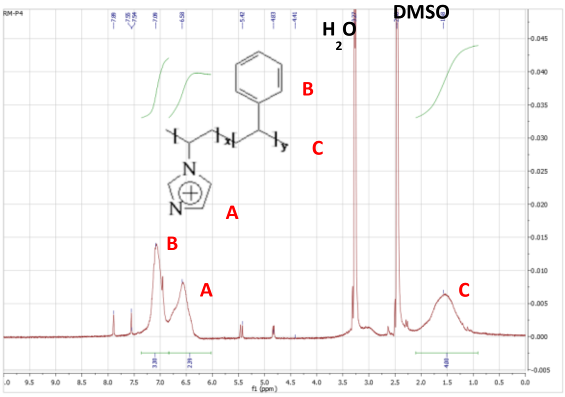

Figure S 2: Typical NMR spectra of the polymers poly-co-styrene-vynilimidazole (P1, P2, P3)

SEM and EDX Imaging

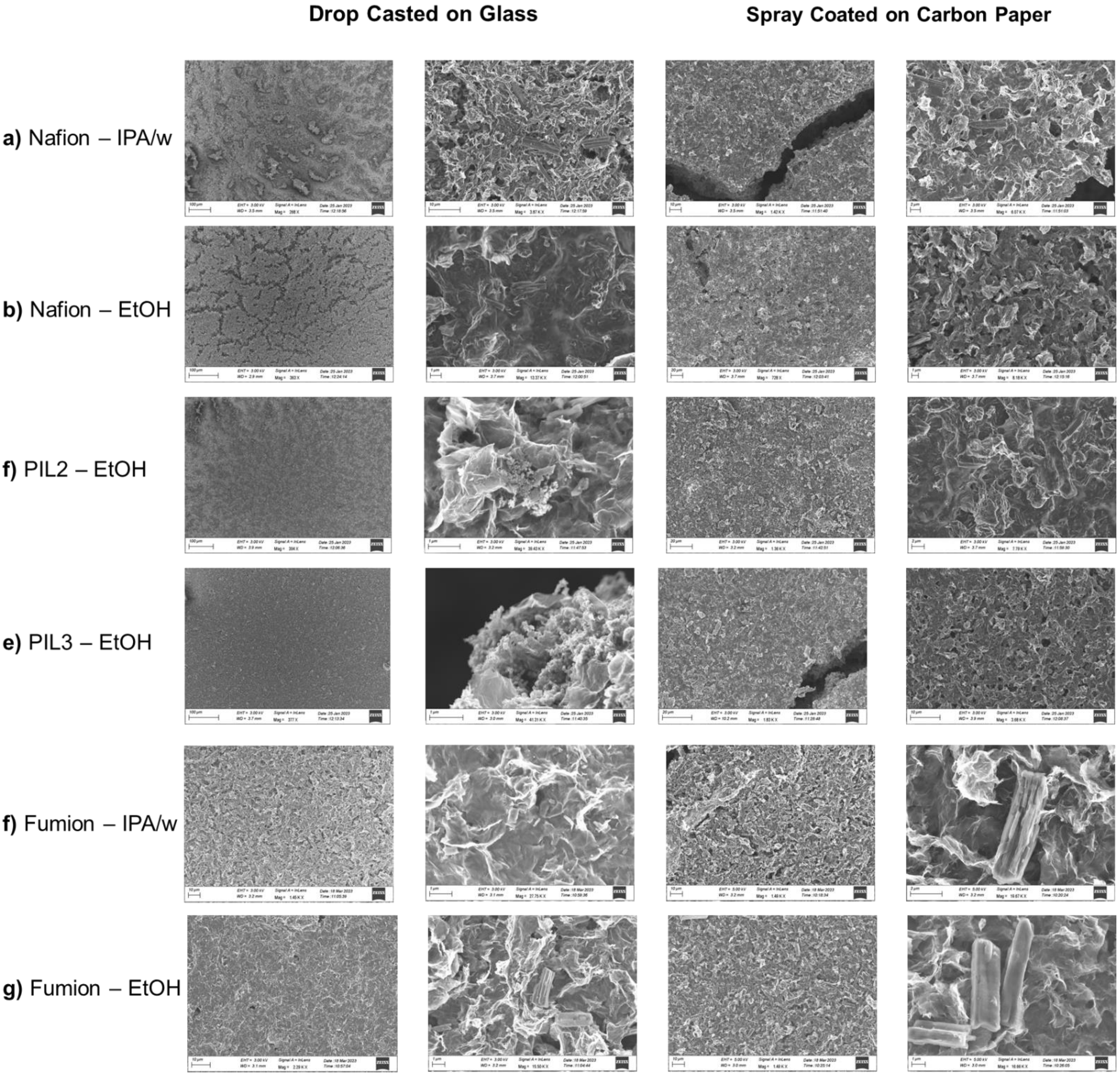

Figure S 3: SEM images of catalyst ink drop casted on a glass substrate (first two columns) or spray coated on carbon paper (third and fourth column).
All the inks contained the following: 4mg/ml FePC/G, ionomer with I/C ratio=1. The solvent used was either ethanol (EtOH) or 50% isopropanol in water (IPA/w). The ionomers compared in this work are the commercially available proton conducting Nafion®, the commercially available anion conductive Fumion®, and two in-house made poly(ionic liquid)s: PIL2 and PIL3

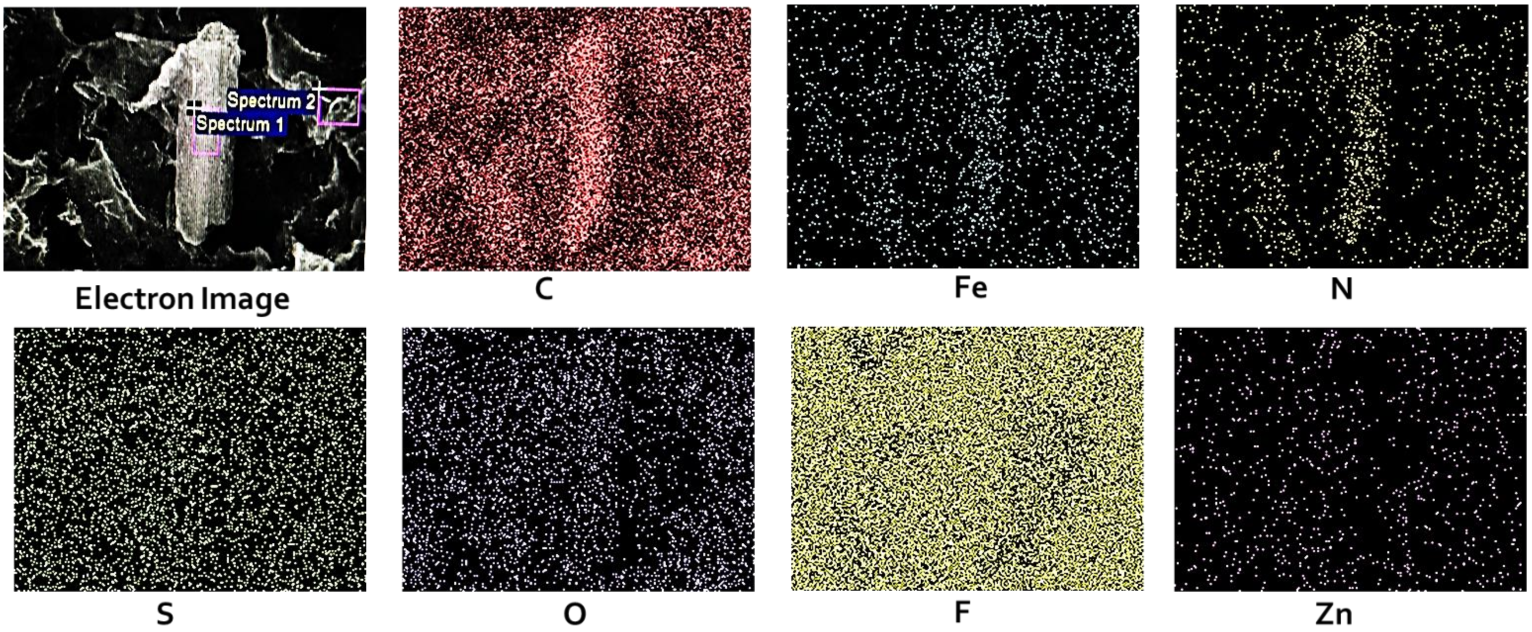

Figure S 4: SEM image and EDX elemental mapping of an electrode area showing cylindrical aggregate typical of the Nafion and Fumion samples.
These structures are believed to originate from FePC aggregation, due to the higher iron and nitrogen content. This particular image was taken on the sample Nafion – IPAw – deposited on glass

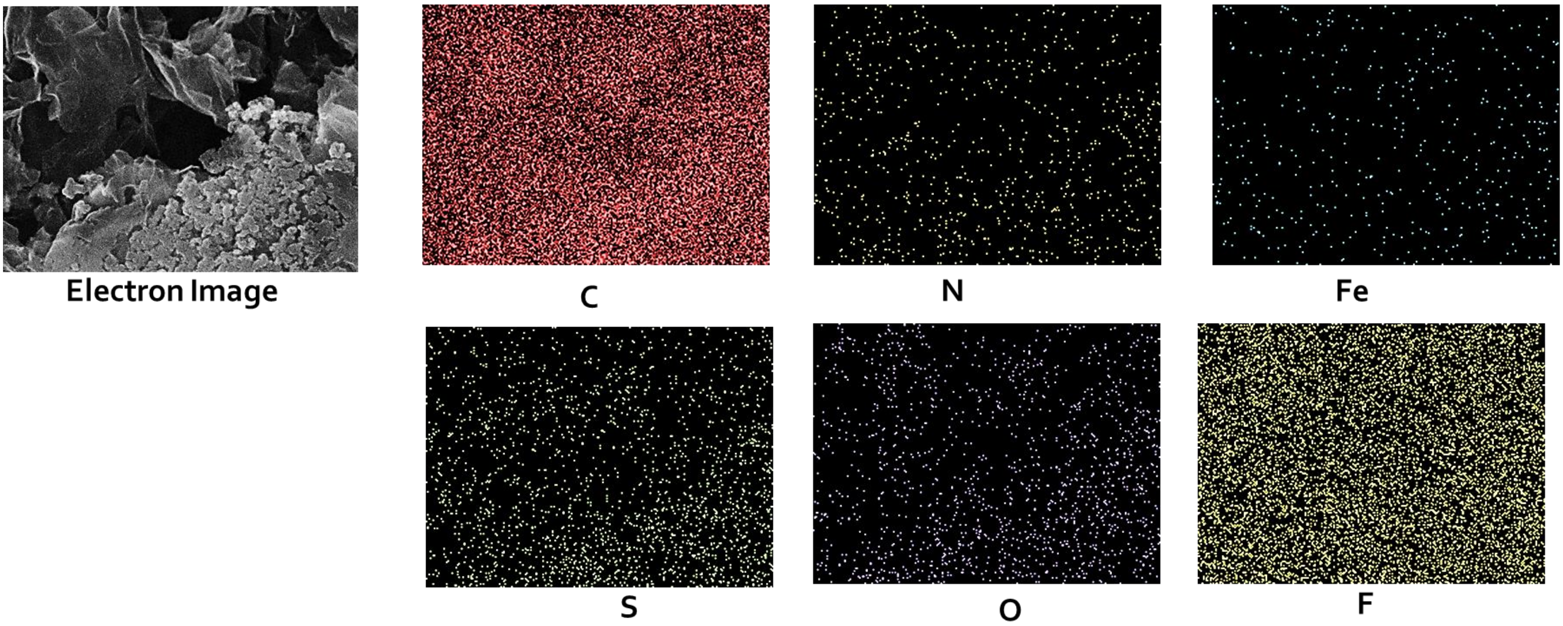

Figure S 5: SEM image and EDX elemental mapping of an electrode area showing round aggregate typical of the PIL2 and PIL3 samples drop casted on glass.
The aggregates are believed to be composed mainly by the ionomer, due to the higher sulphur and oxygen content. This particular image was taken on the sample PIL2 – EtOH – deposited on glass

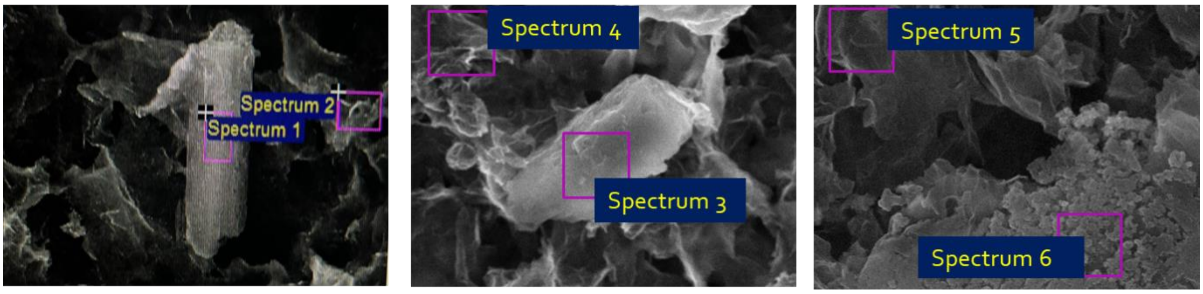

Figure S 6: SEM image and EDX elemental mapping of an electrode area showing the cyclindrical aggregates typical of the Fumion and Nafion samples, and the round aggregate typical of the PIL2 and PIL3 samples. The elemental compositions at the location shown in the SEM images are summarized in table S1
These particular images, from left to right, were taken on the samples: Nafion – IPA on glass (left), Nafion – IPA on carbon paper (middle), PIL2 – EtOH on glass

Table S1: elemental composition at the locations shown in Figure S6, as determined by EDX

**Position**	**C (%w)**	**N (%w)**	**O (%w)**	**F (%w)**	**S (%w)**	**Fe (%w)**
1	50.66	**7.18**	3.94	33.11	0.63	**2.87**
2	45.39	**-**	4.88	45.73	1.29	**-**
3	61.01	**12.27**	4.12	16.02	0.92	**5.49**
4	49.30	**7.00**	2.60	37.19	1.44	**2.47**
5	72.0	-	6.1	**20.1**	**1.7**	**-**
6	70.9	-	3.8	**24.5**	**0.6**	**-**

Catalyst Layer Morphology – Rheology and Particle Size

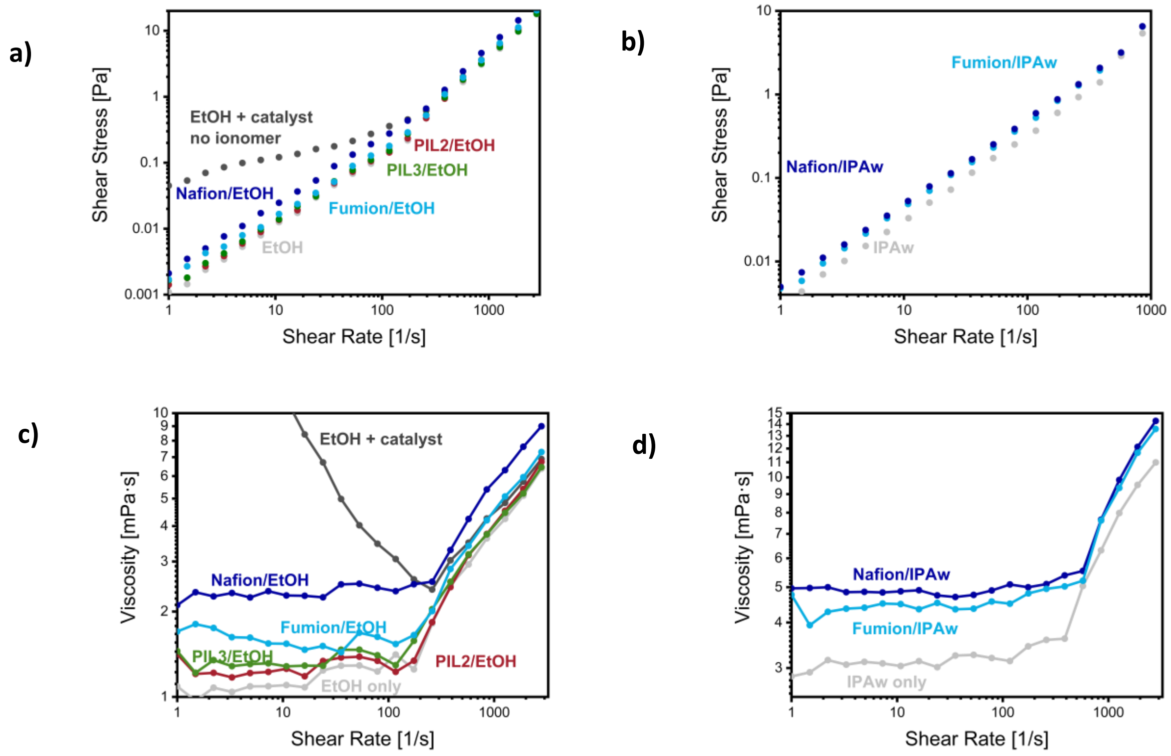

Figure S 7: Rheology of the catalyst inks.
All the inks contain 4mg/ml FePC/G catalysts, with various ionomers in a I/C ratio of 1. The solvent is either ethanol of 50%w isopropanol in water a) Shear stress as a function of shear rate at low shear rates, for the inks in ethanol, b) Shear stress as a function of shear rate at low shear rates, for the inks in 50% isopropanol in water c) Viscosity as a function of shear rate in ethanol d) viscosity as a function of shear rate in 50% IPA/water

**Solvent**	**Ionomer**	**Average Hydrodynamic radius**	**Average polydispersity**
IPA/w	Nafion	745	26
	Fumion	710	25
EtOH	Catalyst only	1118	23
	Nafion	488	25
	Fumion	457	26
	PIL2	500	21
	PIL3	441	21

Table S2: Hydrodynamics radius and polydispersity of the aggregates in the catalyst ink, measured with dynamic light scattering and averaged over at least 3 repeats of 10 runs each. It should be noticed that since the polydispersity index is high, the average diameter can be overestimated,[1] therefore these results should only be taken as a qualitative indication of the catalyst ionomer interaction.

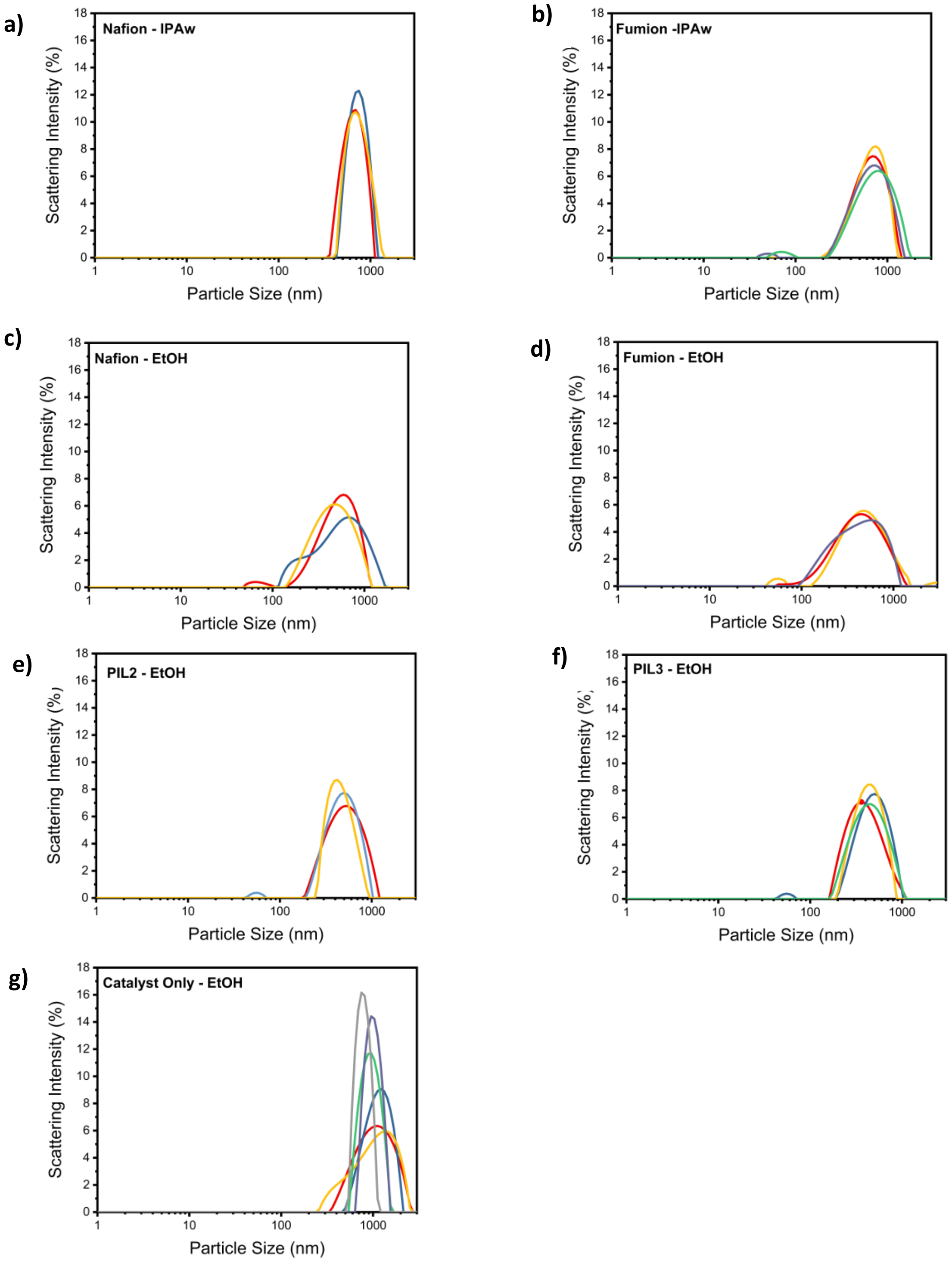

Figure S 8: Dynamic light scattering of the catalyst inks, diluted 100 times.
After dilution, the catalyst inks contained 0.04mg of catalyst and 0.04mg of ionomer per mL of ink. The solvent was either ethanol or 50% isopropanol in water.

Electrochemical Results

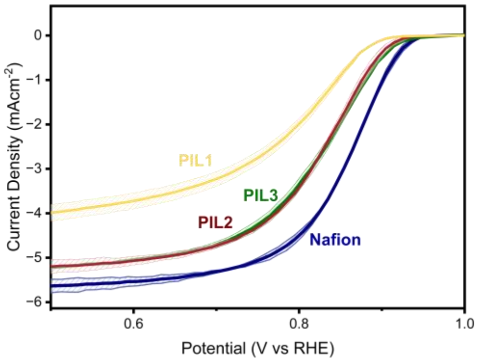

Figure S 11: Comparison of the ORR performance of Nafion and the poly(ionic liquid)s ionomers.
Linear sweep voltammogram obtained in oxygen-saturated 0.1M KOH, at a rotational speed of 1600 rpm and scan rate of 10 mV/s. The catalyst is FePC/G and the ionomer are shown in the figure (Nafion shown in blue, PIL1 is yellow, PIL2 in red and PIL3 in green). The I/C ratio is 1 for all the samples.

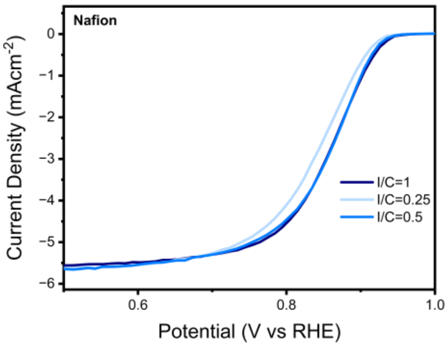

Figure S 9: Optimization of Ionomer to Catalyst ratio for Nafion.
Linear sweep voltammogram obtained in oxygen-saturated 0.1M KOH, at a rotational speed of 1600 rpm and scan rate of 10 mV/s. The catalyst is FePC/G and the ionomer is Nafion, with the I/C ratio shown in caption

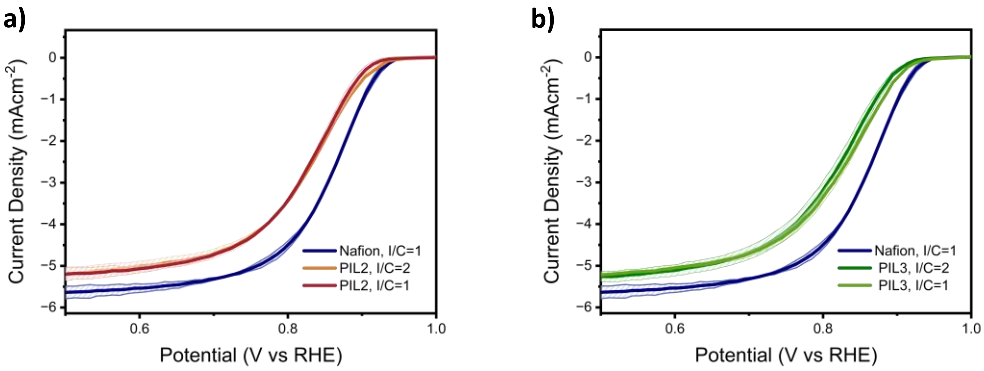

Figure S 10: Optimization of the ionomer to catalyst ratio for the poly(ionic liquid)s ionomers
Linear sweep voltammogram obtained in oxygen-saturated 0.1M KOH, at a rotational speed of 1600 rpm and scan rate of 10 mV/s. The catalyst is FePC/G and the ionomer is and I/C ratio are shown in caption

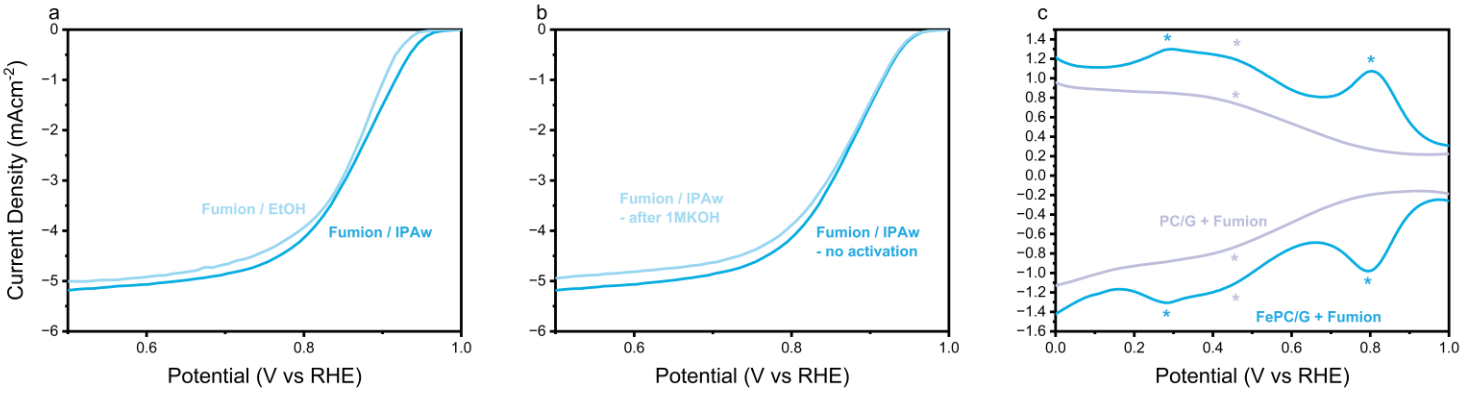

Figure S 12: Further electrochemical studies of Fumion ionomer

a) Linear sweep voltammogram obtained in oxygen-saturated 0.1M KOH, at a rotational speed of 1600 rpm and scan rate of 10 mV/s. The catalyst is FePC/G and the ionomer is Fumion, the I/C ratio is 1 for both samples. The samples differ in the choice of solvent for the ink: 50% IPA in water (darker curve) and ethanol (lighter curve)

b) Linear sweep voltammogram obtained in oxygen-saturated 0.1M KOH, at a rotational speed of 1600 rpm and scan rate of 10 mV/s. The catalyst is FePC/G and the ionomer is Fumion, the I/C ratio is 1 for both samples and the solvent for the catalyst ink is 50% IPA in water. The samples differ for the ionomer activation: the darker curve records the activity of the catalyst layer as prepared, while the lighter curve represents the same RDE electrode after soaking it 1h in 1M KOH

c) Square wave voltammograms obtained in nitrogen-saturated 0.1M KOH, in static conditions, with a frequency of 2Hz, with 4mV steps and a modulation amplitude of 20mV. The catalyst is FePC/G (darker curve) and PC/G (lighter curve). The ionomer is Fumion, the solvent of choice for the catalyst ink is 50% IPA in water and the I/C ratio is 1.

Note on ink solvent

The effect of solvent was investigated for Fumion and Nafion, which were tested in both 50% IPA/water and pure ethanol. Normally, a combination of alcohol and water is used in the ink, but the hereby synthesised polymers were insoluble in water or isopropanol, so pure ethanol was used instead. The same solvent was studied for Fumion and Nafion. The choice of solvent was found to have a small effect on the activity of the catalyst layer, but for the case of Fumion and Nafion, ethanol-based inks were found to be particularly challenging to deposit and results were less reproducible. Therefore, results with IPA/water solvents are shown in the manuscript. Figure S 12a shows a comparison of the activity of FePC/G with Fumion, in the case of IPA/water and ethanol solvent

Note on Fumion activation

Anion exchange ionomer are commonly delivered with a halogenated anion and should undergo ion exchange before use. In the case of Fumion, the ionomer comes in the brominated form and ion exchange usually happen by immerging the deposited catalyst layer in 1M KOH for 1 to 24 hours. In this work, we found that the activity of the catalyst layer does not improve after this ion exchange step either for the case of catalyst drop casted on an RDE electrode or spray coated on carbon paper and tested in GDE. For example, Figure S 12b shows the activity of the same RDE electrode before and after immersing it for 1h in 1M KOH. After soaking the electrodes in 1M KOH overnight the activity dropped slightly, possibly indicating that the ionomer is not stable in alkaline conditions. Therefore all the results shown in the manuscript were obtained after 1h KOH activation.

Note on Fumion CV peaks

As it can been seen in Figure 7d in the manuscript, the cyclic voltammogram of FePC containing Fumion shows a broader peak shifted to higher potential, compared to other ionomers. To further understand the change in shape of this peak, we recorded square wave voltammograms (SWV). Compared to a common CV, in SWV the potential is stepped up and down in every step, allowing to reduce the capacitative contribution and to isolate peaks originating from faradaic processes. The results are shown in Figure S 12c. As it can be observed, the low potential peak is actually composed of two separate peaks. Since this extra peak was not observed for any other ionomer, we hypothesise that it originates from the ionomer. To confirm this hypothesis, we repeated the experiment with the same catalyst without iron, for which no CV peak is normally observed. The PC catalyst with Fumion presented a broad peak at around 0.45V vs RHE, which again was no observed in the absence of Fumion. This confirms that the peak is likely originate from an electron transfer at the ionomer.

Note on Impedance

There is still debate regarding the best fitting for impedance data collected with a rotating disc electrode. However, there is general consensus that the high frequency semicircle can be modelled using a Randel circuit, composed of a polarization resistance (Rp), double layer capacitance (Cdl) and uncompensated resistance (Ru).[2] The uncompensated resistance, or known as ohmic drop, represents the ohmic resistance of the electrolyte between the reference and the working electrode, while Cdl represents the charging and discharging of the electric double layer at the surface of the electrode. The main component of the polarization resistance is the charge transfer resistance, which is related to the kinetics of heterogeneous electrochemical processes. The semicircle present at lower frequency is generally associated with oxygen transport. This can either be represented by a simple resistance (Ro),[3,4] or by a Warburg element (Zo) for the case of a transmissive boundary [2].

$$Z_O = \frac{1}{Y_O\sqrt{j\omega}}\tanh\left(B\sqrt{j\omega}\right), \quad R_O = \frac{B}{Y_O}, \quad D_O = \left(\frac{\delta}{B}\right)^2 \qquad where\ \delta = 1.612D^{\frac{1}{3}}\nu^{1/6}/\sqrt{\omega}$$

Finally, Co represents the capacitance originating from the diffusion process. Rather than using pure capacitance elements, we used constant phase elements for the fitting. Deviation from the ideality of capacitance is typical of porous electrodes. [2]

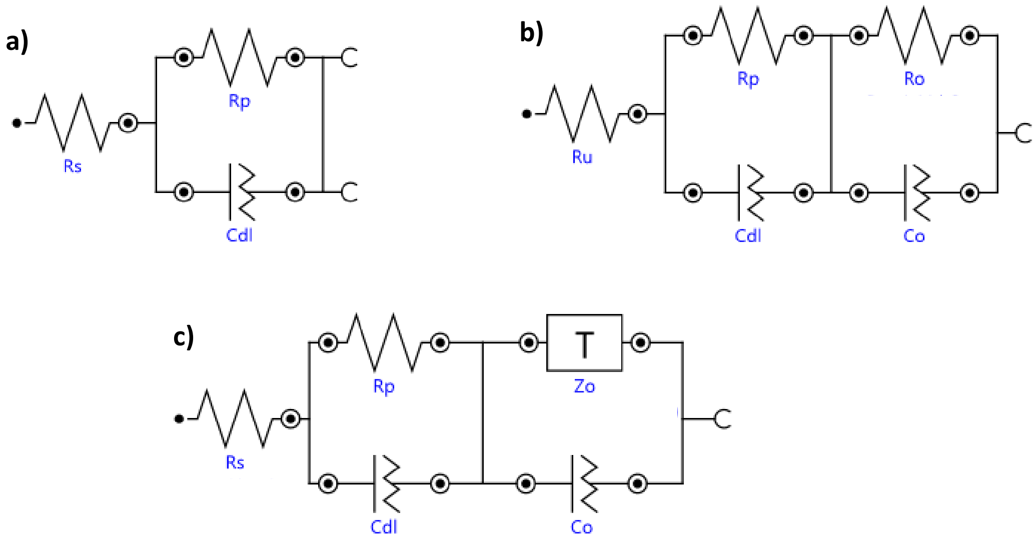

Figure S 13: Equivalent circuit for fitting of Impedance spectroscopy measurements

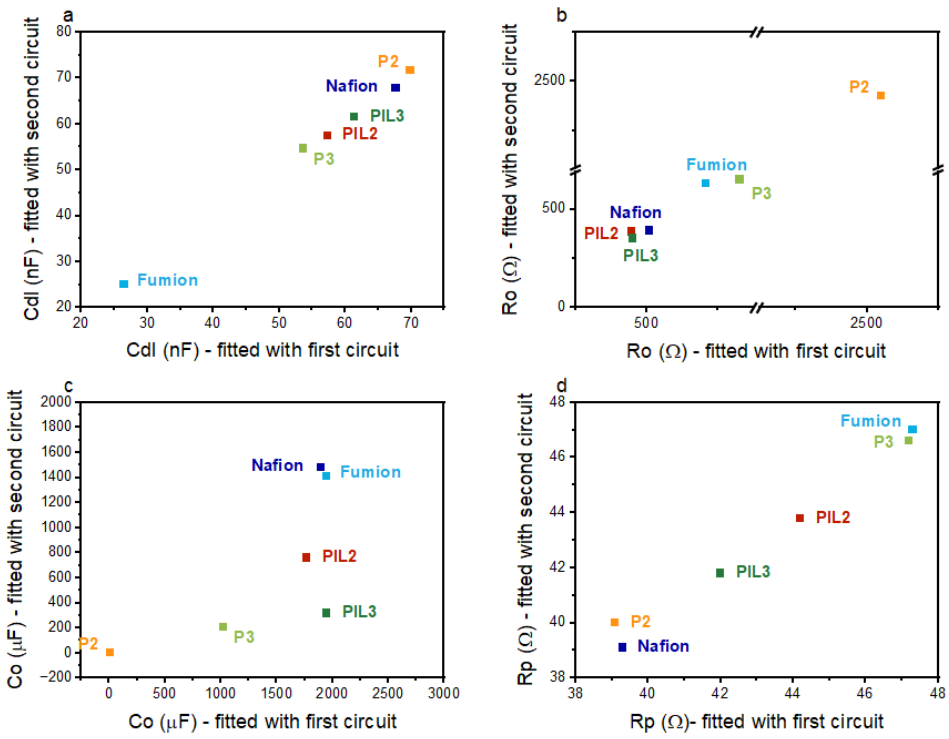

Figure S 14: Comparison of Impedance fitting parameters obtained with the equivalent circuits in Figure S 13b (first circuit) and Figure S 13 (second circuit).

	Rp1 (Ω)	Rp2 (Ω)	Cdl1 (nF)	Cdl2 (nF)	Ro1 (Ω)	Ro2 (Ω)	Co1 (μF)	Co2 (μF)
**Nafion**	39.3	39.1	67.7	67.8	509	391	1900	1480
**Fumion**	47.3	47	26.5	25.1	707	631	1950	1410
**P2**	39.1	40	69.9	71.7	2550	2426	12.2	3.14
**PIL2**	44.2	43.8	57.3	57.5	448	385	1770	760
**P3**	47.2	46.6	53.6	54.6	826	650	1020	204
**PIL3**	42	41.8	61.4	61.5	450	352	1950	318

Table S 3: Results of the fitting obtained with the equivalent circuits in in Figure S 13b (first circuit) and Figure S 13 (second circuit).

Study of loading effect

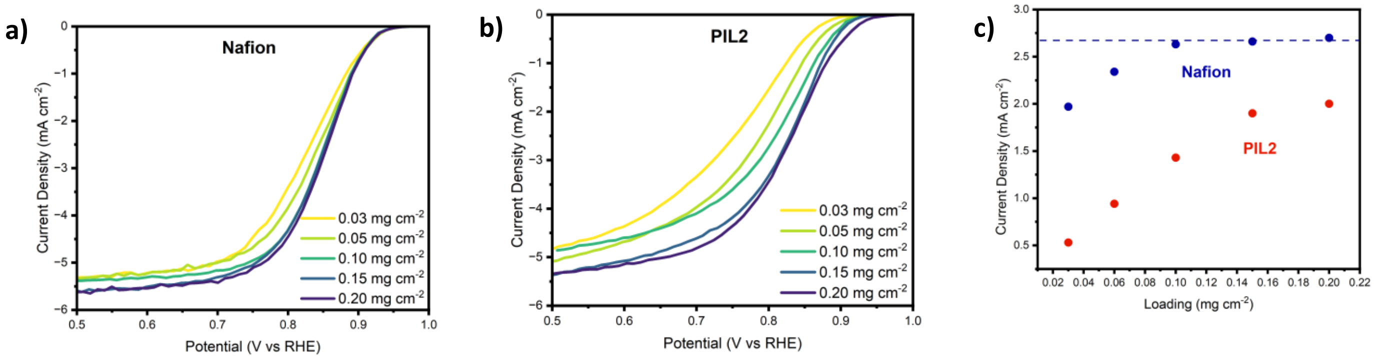

Figure S 15: Effect of catalyst loading on the oxygen reduction performance. a) shows the oxygen reduction performance of FePC/G with Nafion ionomer, at the loadings indicated in the legend. b) shows the same results, when using PIL2 as an ionomer. For all the measurements, an I:C ratio of 1 was used. c) shows the current density at 0.85V vs RHE as a function of the catalyst loading

Optical Microscopy

Nafion

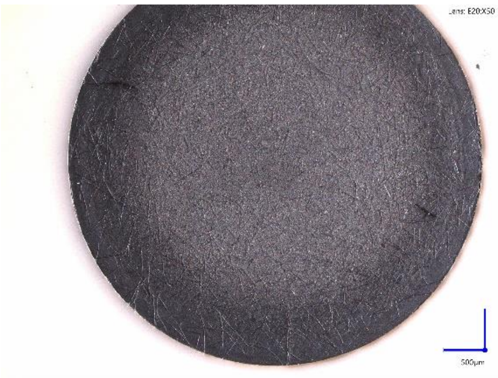

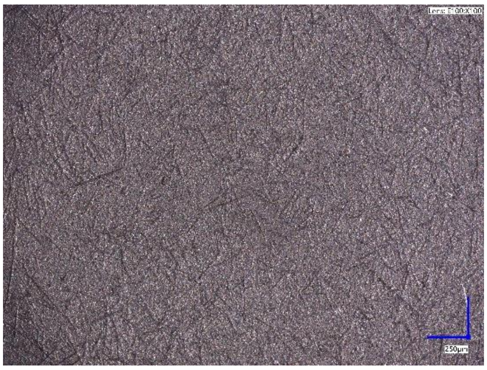

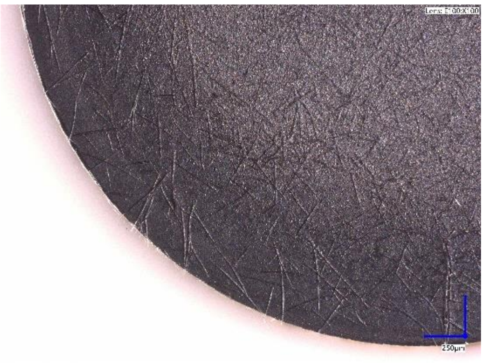

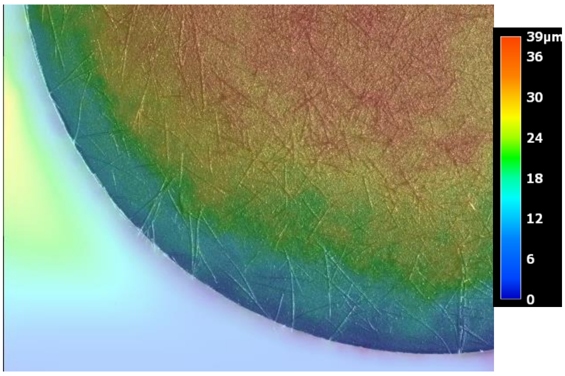

PIL2

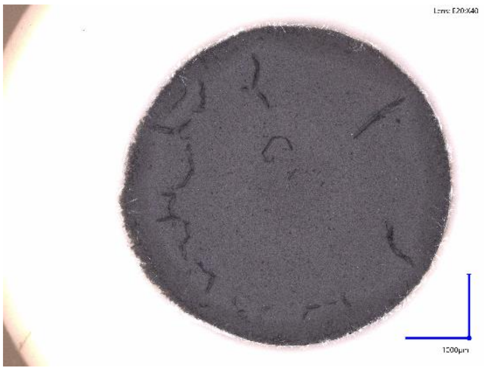

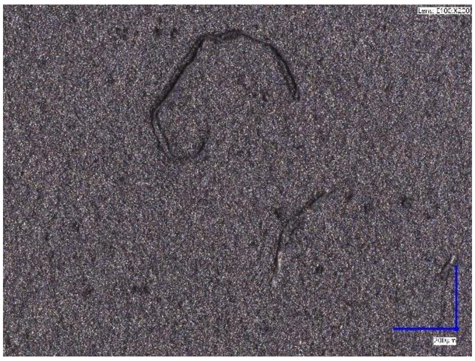

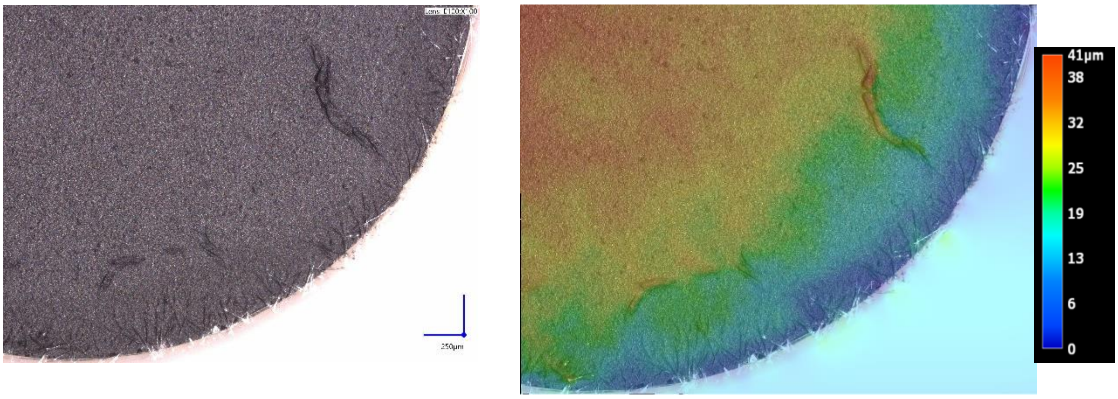
250µm
41µm
38
32
25
19
13
6
0

PIL3

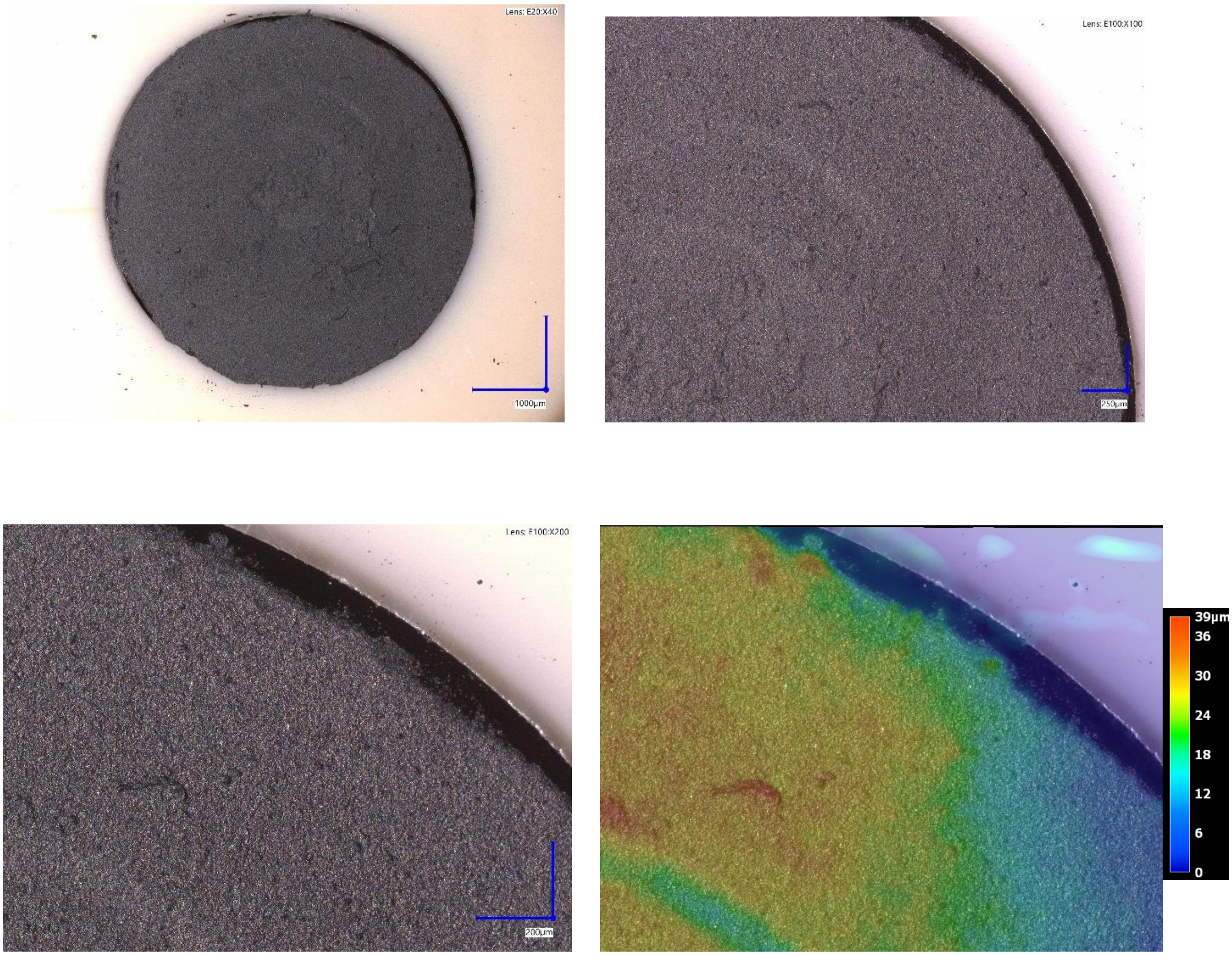

Figure S 16: Optical microscopy images of the RDE electrode with a drop-casted layer of 0.14 mg cm $^{-2}$ FePC/G with the ionomers Nafion, PIL2 and PIL3, with an I:C ratio of 1. All the inks contained the following: 4mg/ml FePC/G, ionomer with I/C ratio=1. The solvent used was ethanol for PIL2, PIL3 and 50% isopropanol in water for Nafion.

References

(1) Anderson, W.; Kozak, D.; Coleman, V. A.; Jämting, Å. K.; Trau, M. A Comparative Study of Submicron Particle Sizing Platforms: Accuracy, Precision and Resolution Analysis of Polydisperse Particle Size Distributions. *J. Colloid Interface Sci.* **2013**, *405*, 322–330. https://doi.org/10.1016/j.jcis.2013.02.030.

(2) Ch. Lazanas, A.; I. Prodromidis, M. Electrochemical Impedance Spectroscopy–A Tutorial. *ACS Meas. Sci. Au* **2023**, *0* (0). https://doi.org/10.1021/acsmeasuresciau.2c00070.

(3) Ruiz-Camacho, B.; Baltazar Vera, J. C.; Medina-Ramírez, A.; Fuentes-Ramírez, R.; Carreño-Aguilera, G. EIS Analysis of Oxygen Reduction Reaction of Pt Supported on Different Substrates. *Int. J. Hydrogen Energy* **2017**, *42* (51), 30364–30373. https://doi.org/10.1016/j.ijhydene.2017.08.087.

(4) Singh, R. K.; Devivaraprasad, R.; Kar, T.; Chakraborty, A.; Neergat, M. Electrochemical

Impedance Spectroscopy of Oxygen Reduction Reaction (ORR) in a Rotating Disk Electrode Configuration: Effect of Ionomer Content and Carbon-Support. *J. Electrochem. Soc.* **2015**, *162* (6), F489–F498. https://doi.org/10.1149/2.0141506jes.
